# Sleep Problems Associate With Multimorbidity: A Systematic Review and Meta-analysis

**DOI:** 10.3389/phrs.2023.1605469

**Published:** 2023-06-13

**Authors:** Yaguan Zhou, Yichen Jin, Yi Zhu, Weiwei Fang, Xiaochen Dai, Carmen Lim, Shiva Raj Mishra, Peige Song, Xiaolin Xu

**Affiliations:** ^1^ School of Public Health and the Second Affiliated Hospital, Zhejiang University School of Medicine, Hangzhou, China; ^2^ School of Public Health, Tongji Medical College, Huazhong University of Science and Technology, Wuhan, China; ^3^ Shulan International Medical College, Zhejiang Shuren University, Hangzhou, China; ^4^ Department of Health Metrics Sciences, School of Medicine, University of Washington, Seattle, WA, United States; ^5^ National Centre for Youth Substance Use Research, Faculty of Health and Behavioral Sciences, The University of Queensland, Brisbane, QLD, Australia; ^6^ School of Psychology, Faculty of Health and Behavioral Sciences, The University of Queensland, Brisbane, QLD, Australia; ^7^ Academy for Data Science and Global Health, Bharatpur, Nepal; ^8^ School of Public Health and Women’s Hospital, Zhejiang University School of Medicine, Hangzhou, China; ^9^ School of Public Health, Faculty of Medicine, The University of Queensland, Brisbane, QLD, Australia

**Keywords:** multimorbidity, sleep duration, aging, insomnia, sleep problems

## Abstract

**Objectives:** To summarize the evidence on the association between sleep problems and multimorbidity.

**Methods:** Six electronic databases (PubMed, Web of Science, Embase, China National Knowledge Infrastructure, VIP, and Wan fang) were searched to identify observational studies on the association between sleep problems and multimorbidity. A random-effects model was used to estimate the pooled odds ratios (ORs) and 95% confidence intervals for multimorbidity.

**Results:** A total of 17 observational studies of 133,575 participants were included. Sleep problems included abnormal sleep duration, insomnia, snoring, poor sleep quality, obstructive sleep apnea (OSA) and restless legs syndrome (RLS). The pooled ORs (95% CIs) for multimorbidity were 1.49 (1.24–1.80) of short sleep duration, 1.21 (1.11–1.44) of long sleep duration and 2.53 (1.85–3.46) for insomnia. The association of other sleep problems with multimorbidity was narratively summarized due to limited number of comparable studies.

**Conclusion:** Abnormal sleep duration and insomnia are associated with higher odds of multimorbidity, while the evidence on association of snoring, poor sleep quality, obstructive sleep apnea and restless legs syndrome with multimorbidity remains inconclusive. Interventions targeting sleep problems should be delivered for better management of multimorbidity.

## Introduction

With the development of society and the change of lifestyle, sleep behaviors altered substantially in daily life [1]. Sleep problems, including abnormal sleep duration, insomnia, snoring, obstructive sleep apnea (OSA) and so on, have been estimated to affect a large proportion of the global population [2–6]. Individuals with sleep problems experienced increased risks of chronic conditions, including stroke [7,8], heart failure [9], asthma [10] and dementia [11]. Recently, the increasing prevalence of chronic conditions among adults especially the elders [12] leads to considerable interest in the association between sleep problems and multiple chronic conditions (multimorbidity).

Multimorbidity was usually defined as the coexistence of two or more chronic conditions, which has also become an important public health concern [13]. The prevalence of multimorbidity increased by age, which was nearly 100% in older participants [14]. Multimorbidity is associated with disability, functional limitations, higher healthcare expenditure and increased mortality, posing a persistent burden on global healthcare systems [15, 16].

Previous systematic reviews and meta-analyses have observed the association between sleep problems and a series of health outcomes. For example, the significant association was found between sleep duration and several chronic conditions, including mortality, diabetes, cardiovascular disease (CVD), coronary heart disease, and obesity [17, 18]. Insomnia was also proven to relate with mental disorders, including depression [19], anxiety, alcohol abuse, and psychosis [20], as well as cognitive decline [21]. Another systematic review and meta-analysis of 22 studies found sleep quality was positively associated with metabolic syndrome [22]. However, no systematic reviews of the association between sleep problems and multimorbidity exist.

Given an increasing burden of chronic conditions and multimorbidity was observed among population with sleep problems [23], addressing the knowledge gap on this topic may have important implications for individuals, healthcare systems, and society. In the present study, we conducted a systematic review and meta-analysis to examine the association between sleep problems and multimorbidity.

## Methods

### Search Strategy and Data Sources

This systematic review and meta-analysis were conducted in accordance with the PRISMA 2020 statement [24]. Six electronic databases, including PubMed, Embase, Web of Science, China National Knowledge Infrastructure, VIP, and Wan fang, were searched from inception to November 2021. The search strategy included both MeSH terms and free words referring to sleep problems and multimorbidity. Considering some primary studies did not distinguish multimorbidity from comorbidity (the presence of additional diseases in relation to an index disease in one individual), the term “comorbidity” was also included in the search strategies, while studies truly focusing on comorbidity (the presence of additional diseases in relation to an index disease in one individual) would further be excluded. Details of the searching strategy are included in Supplementary Tables S1–S3. Reference lists of the included studies and systematic reviews reporting on the same or related topic were also manually scanned. No unpublished data was used in our study.

### Studies Selections and Data Extraction

Two reviewers (YaZ and YJ) independently screened the title and abstract of selected studies, and further read the full text for inclusion. We included observational studies (cohort studies, cross-sectional studies, or case-control studies) focusing on the association between any sleep problems and multimorbidity among adults. We excluded studies focusing on sleep problems and its comorbidity, and whose target population was children or adolescents. A language restriction of English and Chinese was applied. Any disagreements were resolved through consultation with a third investigator (XX).

After identifying eligible studies, YaZ and YJ independently extracted information from each study using pre-designed data extraction forms. The following items of information were manually extracted: first author, publication year, journal, study title, study design, country, sample size, population age, the proportion of males, the definition and classification of sleep problems, follow-up length (for cohort studies), the definition of multimorbidity, statistical methods, and effect sizes. Any disagreement between the two reviewers regarding the data extraction process was resolved through discussion with XX.

### Quality Assessment

The 11-item checklist recommended by the Agency for Healthcare Research and Quality (AHRQ) and the Newcastle-Ottawa scale (NOS) were used to evaluate the methodological quality of cross-sectional studies and cohort studies, respectively (Supplementary Table S4). In the AHRQ assessment checklist, every item has three response options: yes, no, and unclear. Each item scores one point. Cross-sectional studies with scores of 0–3, 4–7, and 8–11 were recorded as low, moderate, and high-quality studies, respectively [25]. For cohort studies, the NOS allocates a maximum of nine points for the quality of study selection (0–4 points), the comparability of the groups (0–2 points), and the ascertainment of the outcome (0–3 points). Cohort studies with points of 0–3, 4–6, and 7–9 were deemed as low, moderate, and high quality, respectively [26]. Two reviewers (YaZ and YJ) independently conducted the quality assessment and discrepancies were resolved in consensus.

### Data Analysis

We narratively described the findings of included studies, and conducted meta-analyses for sleep problems which were reported by at least three comparable studies. Considering the potential heterogeneity between studies, a random-effects model was used to pool the odds ratios (ORs) and 95% confidence intervals (CIs) for the association between sleep problems and multimorbidity. The generic inverse variance method was used to assign weights to each study. Studies with a more precise estimate of the effect size have low variance and are assigned more weight, and those with a less precise estimate of the effect size have high variance and are assigned less weight [27]. Cochran’s Q test and the I^2^ statistic were used to indicate heterogeneity between studies for each meta-analysis, with the former considering *p* < 0.05 as significant for heterogeneity, and the latter having cut-offs of 25%, 50%, and 75% for low, medium, and high heterogeneity [25], respectively. Meta-regression analyses and subgroup analyses were conducted to determine any valid sources of heterogeneity and between-study differences. Publication year, income country type, population age, and the definition of sleep problems and multimorbidity were included in the meta regression analyses as the independent variables. Moreover, we identified the presence of outliers whose CIs had no overlap with that of the pooled effect size, and repeated meta-analyses after excluding outliers. Visual inspection of funnel plots and the Egger’s regression test were used to assess publication bias.

Additional meta-analyses were conducted to assess the association between sleep problems and several chronic conditions which were reported by at least three comparable studies.

All analyses were performed in Review Manager Version 5.3 (Copenhagen, Denmark: The Nordic Cochrane Centre, The Cochrane Collaboration) and Stata MP version 17.0 (College Station, TX).

## Results

### Search Results

**FIGURE 1:**
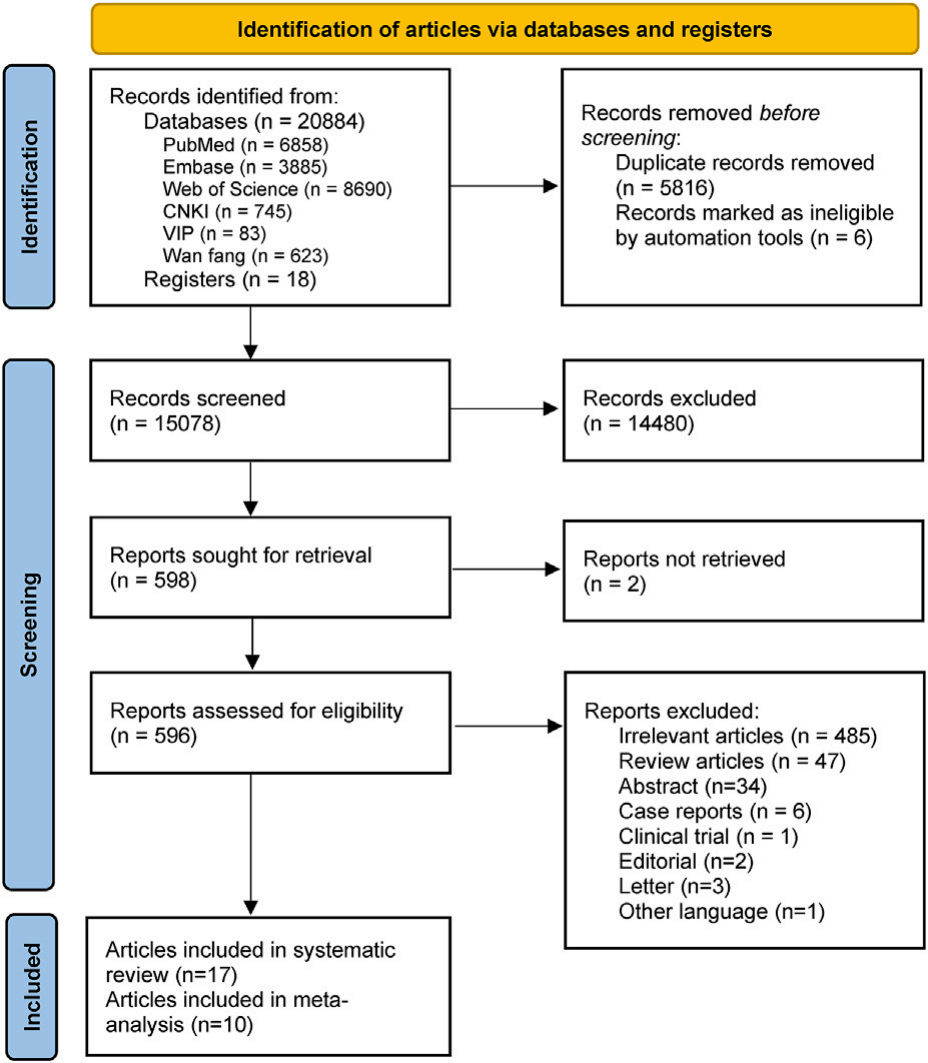
Flowchart of study selection (Australia, Brazil, Canada, China, England, Germany, Hungary, Italy, Luxemburg, Portugal, Scotland, and Wales. 2006–2021).

The initial search yielded a total of 20,902 studies. After removing duplicates and screening the titles and abstracts, 596 full-text studies were assessed for eligibility. Based on the eligible criteria, 17 observational studies were finally included in this systematic review, with a total of 133,575 participants [16, 28–43] (Figure 1). The reasons for study exclusion are provided in Supplementary Table S5.

### Study Characteristics

The included studies were published between 2005 and 2021, with the sample size ranging from 120 [35] to 30,011 [34]. Most studies (*N* = 15) were cross-sectional studies [16, 28, 30–38, 40–43], and the remaining two were cohort studies (one included analyses of both cross-sectional and longitudinal data) [29,39]. The included 17 studies were conducted in China [29, 33, 41–43], Australia [28, 36], Germany [30, 39], Canada [34, 35], Italy [31], Brazil [32], Portugal [16], Luxemburg [37], the United Kingdom [38], and Hungary [40]. The majority of studies (*N* = 15) were published in English [16, 28–32, 34–42], and two were in Chinese [33, 43]. Detailed characteristics of included studies as well as the definition of sleep problems and multimorbidity are provided in Table 1; Supplementary Table S6.

**TABLE 1:** Characteristics of cross-sectional and cohort studies of sleep problems with multimorbidity (Australia, Brazil, Canada, China, England, Germany, Hungary, Italy, Luxemburg, Portugal, Scotland, and Wales. 2006–2021).

Study	Location, study design	Sleep problems	Multimorbidity (vs. References)
Appleton et al. [28]	Australia, Cross-sectional	OSA	2 or more chronic conditions (vs. 0 chronic conditions)
		Insomnia	
		RLS	
		Snoring	
He et al. [29]	China, Cohort	Sleep duration	2 or more chronic conditions (vs. 0/1 chronic condition)
Helbig et al. [30]	Germany, Cross-sectional	Insomnia	2 or more chronic conditions (vs. 0/1 chronic condition)
		Sleep duration	
Lacedonia et al. [31]	Italy, Cross-sectional	OSA	3 or more chronic conditions (vs. 0–2 chronic conditions)
Lima et al. [32]	Brazil, Cross-sectional	Sleep duration	3 or more chronic conditions (vs. 0 chronic conditions)
Liu et al.[33]	China, cross-sectional	Sleep quality	2 or more chronic conditions (vs. 0/1 chronic condition)
Nicholson et al. [34]	Canada, Cross-sectional	Sleep duration	2 or more chronic conditions (vs. 0/1 chronic condition)
		Sleep quality	
Reis et al. [16]	Portugal, Cross-sectional	Sleep duration	2 or more chronic conditions (vs. 0/1 chronic condition)
Robichaud-Hallé et al. [35]	North Canada, Cross-sectional	OSA	DBMA 10 (vs. DBMA 0)
Ruel et al. [36]	Australia, Cross-sectional	OSA	2 or more chronic conditions (vs. 0/1 chronic condition)
			3 or more chronic conditions (vs. 0–2 chronic condition)
Ruiz-Castel et al. [37]	Luxemburg, Cross-sectional	Sleep duration	3 or more chronic conditions (vs. 0 chronic conditions)
Stewart et al. [38]	England, Scotland, and Wales, Cross-sectional	Insomnia	2 or more chronic conditions (vs. 0 chronic conditions)
Szentkirályi et al. [39]	Germany, Cohort	RLS	Trend per 1 condition increase
Torzsa et al. [40]	Hungary, Cross-sectional	Snoring	3 or more chronic conditions (vs. 0 chronic conditions)
Wang et al. [41] ^a^	China, Cross-sectional	Sleep duration	2 or more chronic conditions (vs. 0 chronic conditions)
Wanget al. [42] ^a^	China, Cross-sectional	Sleep duration	2 or more chronic conditions (vs. 0 chronic conditions)
Zhanget al. [43]	China, Cross-sectional	Sleep duration	2 or more chronic conditions (vs. 0/1 chronic condition)
		Snoring	

OSA, obstructive sleep apnea; RLS, restless legs syndrome; DBMA, the Disease Burden Morbidity Assessment.

^a^ Wang et al. [41] and Wang et al. [42] were based on the same investigation, but the former one focused on participants aged 18–59 years, and Wang et al. [42] focused on participants aged 60–79 years.

Among the included studies, nine explored the association between abnormal sleep duration and multimorbidity [16, 29, 30, 32, 34, 37, 41–43], while the remaining studies focused on the sleep problems of insomnia (*N* = 3) [28, 30, 38], snoring (*N* = 3) [28, 40, 43], poor sleep quality (*N* = 2) [33, 34], OSA (*N* = 4) [28, 31, 35, 36], and RLS (*N* = 2) [28, 39]. Participants’ information on sleep problems was obtained by self-report, medical records, or objective instruments (e.g., polysomnography, overnight monitoring).

Most studies defined multimorbidity as two or more co-existing chronic conditions [16, 28–30, 33, 34, 38, 41–43], five studies defined it as three or more chronic conditions [31, 32, 37, 40], and one study reported results for both measures of multimorbidity [36]. One study used the Disease Burden Morbidity Assessment (DBMA) to measure multimorbidity, with a score of 10 referring to two or more chronic conditions [35]. One study reported effect sizes for per 1 condition increase [39]. In the list of chronic conditions, ten studies included both physical and mental conditions [16, 28–30, 32, 34, 36, 37, 39, 40], and the remaining seven only included physical conditions [31, 33, 35, 38, 41–43].

According to the AHRQ assessment checklist, 11 cross-sectional studies presented moderate methodological quality [16, 28, 31–34, 38, 40–43], and four cross-sectional studies presented high methodological quality [30, 35–37]. According to the NOS scale, the two cohort studies both presented moderate methodological quality [29, 39]. More details on quality assessment can be found in Supplementary Tables S7, S8.

### Abnormal Sleep Duration and Multimorbidity

**FIGURE 2:**
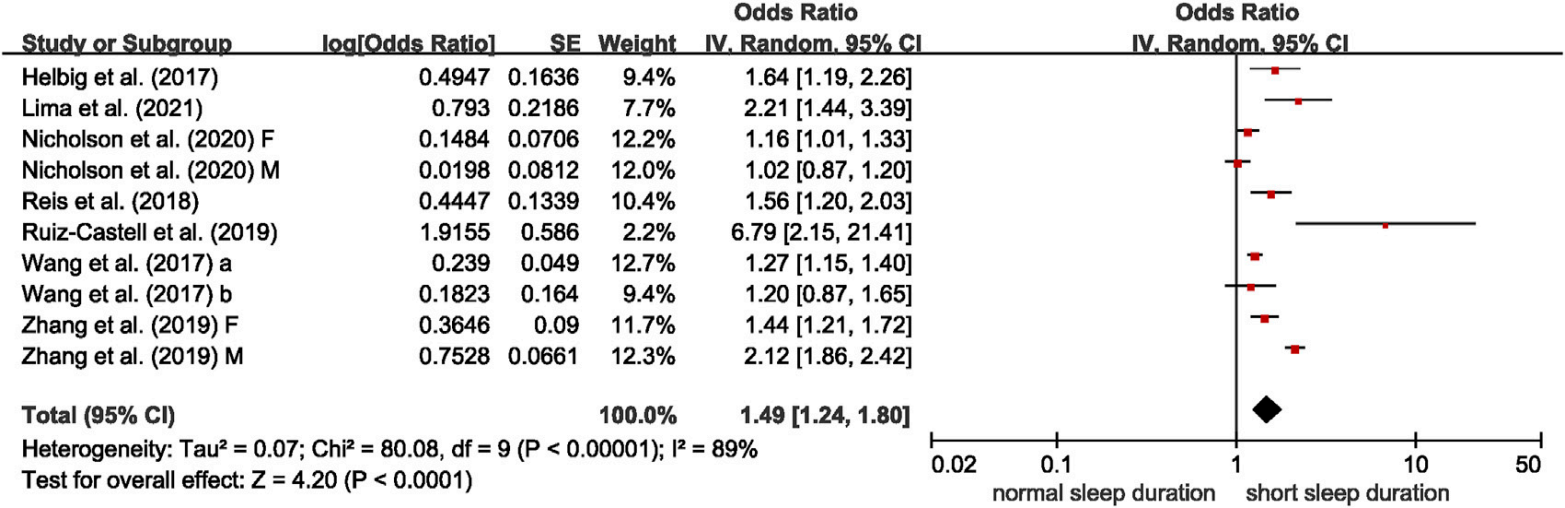
Meta-analysis of the association between short sleep duration and multimorbidity in the adjusted model, derived from available cross-sectional studies (Brazil, Canada, China, Germany, Luxemburg, and Portugal. 2017–2021). Odds ratios (ORs) and 95% confidence intervals (CIs) were derived from original studies. Sleep duration categories (short, normal): Helbig et al. [30]– ≤5 h, 7–8 h; Lima et al. [32]—≤6 h, 7–8 h; Nicholson et al. [34]—<6 h, 6–8 h; Reis et al. [16]—≤5 h, 6–8 h; Ruiz-Castell et al. [37]—<6 h, 6–9 h; Wang et al. [41]—<7 h, 7–9 h; Wang et al. [42]—<7 h, 7–8 h; Zhang et al. [43]—≤6, 8 h.

**FIGURE 3:**
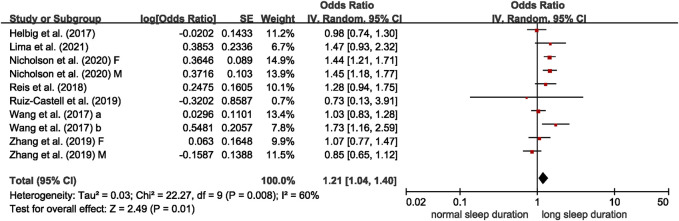
Meta-analysis of the association between long sleep duration and multimorbidity in the adjusted model, derived from available cross-sectional studies (Brazil, Canada, China, Germany, Luxemburg, and Portugal. 2017–2021). Odds ratios (ORs) and 95% confidence intervals (CIs) were derived from original studies. Sleep duration categories (normal, long): Helbig et al. [30]—7–8 h, ≥10 h; Lima et al. [32]—7–8 h, ≥9 h; Nicholson et al. [34]—6–8 h, >8 h; Reis et al. [16]—6–8 h, >9 h; Ruiz-Castell et al. [37]—6–9 h, >9 h; Wang et al. [41]—7–9 h, >9 h; Wang et al. [42]—7–8 h, >8 h; Zhang et al. [43]—8 h, ≥9 h.

Nine studies reported on the association between abnormal sleep duration and multimorbidity, with eight cross-sectional studies entered meta-analysis [16, 30, 32, 34, 37, 41–43]. Results from meta-analyses suggested significant associations of short sleep duration with multimorbidity (OR = 1.49, 95% CI = 1.24–1.80), compared to those with normal sleep duration, with high between-study heterogeneity (I^2^ = 89%, *p* < 0.001) (Figure 2). Long sleep duration was also found to associate with multimorbidity (OR = 1.21, 95% CI = 1.04–1.40), with medium between-study heterogeneity (I^2^ = 60%, *p* = 0.008) (Figure 3). The remaining study of 5,321 Chinese residents aged 45 or more years was not entered the meta-analysis and observed a higher risk of multimorbidity in those with abnormal sleep duration (short or long) after a 4-year follow-up (OR of <7 h or >9 h sleep duration = 1.53, 95% CI = 1.28–1.83) [29].

In the meta-regression analyses, no variables were significant moderators for the association between short sleep duration and multimorbidity. We conducted subgroup analysis by the definition of multimorbidity (b = 0.693, *p* = 0.057 in the meta-regression analyses). The eight cross-sectional studies were stratified into subgroups of “2 or more” and “3 or more,” and both subgroups showed significant association between short sleep duration and multimorbidity (2 or more: OR = 1.39, 95% CI = 1.15–1.67, I^2^ = 90%; 3 or more: OR = 3.40, 95% CI = 1.17–9.89, I^2^ = 69%) (Supplementary Figure S1). For long sleep duration, the definition of long sleep duration contributed significantly to heterogeneity (b = 0.406, *p* = 0.037), and the heterogeneity reduced from 60% to 0% according to meta-regression analysis. The results of subgroup analysis by definition of long sleep duration showed only the group of >8 h/≥8 h observed significant associations with multimorbidity (OR = 1.47, 95% CI = 1.30–1.67, I^2^ = 0%) (Supplementary Figure S1). After excluding three outliers, the effect size was still comparable (OR = 1.46, 95% CI = 1.22–1.75), but the heterogeneity was much lower (I^2^ = 59%) (Supplementary Figure S2).

Funnel plots and the Egger’s regression test indicated no significant publication bias (*p* = 0.415 for short sleep duration; and *p* = 0.649 for long sleep duration) (Supplementary Figure S3).

### Insomnia and Multimorbidity

**FIGURE 4:**
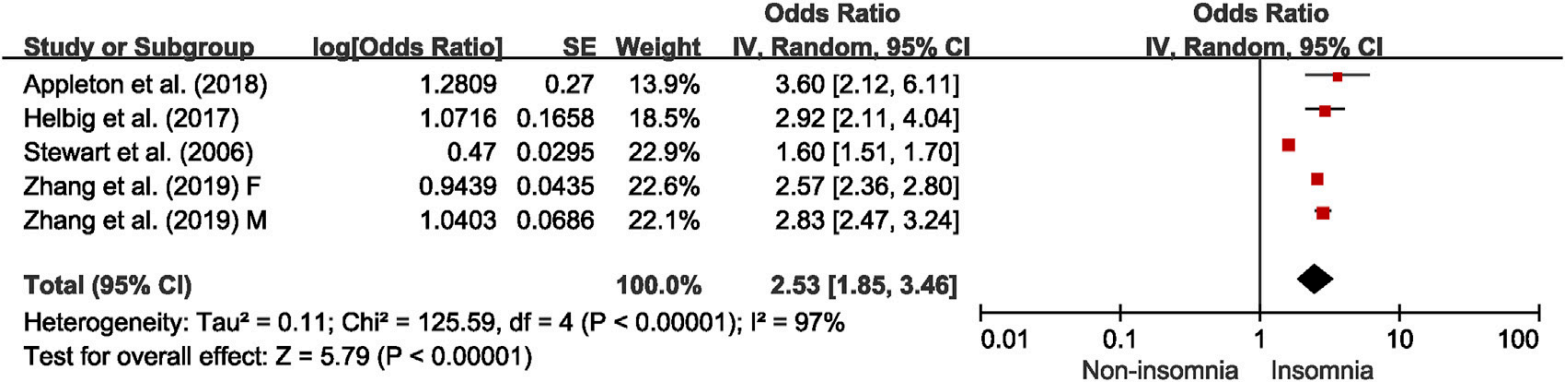
Meta-analysis of the association between insomnia and multimorbidity in the adjusted model, derived from available cross-sectional studies (Australia, China, England, Germany, Scotland and Wales. 2006–2019).

Four studies reporting on the association between insomnia and multimorbidity were meta-analyzed [28, 30, 38]. The association between insomnia and multimorbidity is shown in Figure 4, and the pooled OR was 2.53 (95% CI = 1.85–3.46), with significant between-study heterogeneity (I^2^ = 97%). The results of meta-regression analyses show that the publication years contributed significantly to heterogeneity (b = −0.523, *p* = 0.005, I^2^ from 97% to 0%). The group of recent and early publication years both found significant association between insomnia and multimorbidity (recent: OR = 2.67, 95% CI = 2.49–2.86, I^2^ = 0%; early: OR = 1.60, 95% CI = 1.51–1.70) (Supplementary Figure S4). When one outlier was removed, the pooled OR for the association between insomnia and multimorbidity was 2.67 (95% CI = 2.49–2.86), with no heterogeneity (Supplementary Figure S5). Funnel plots and the Egger’s regression test indicated no significant publication bias (*p* = 0.294) (Supplementary Figure S6). Specially, Stewart et al. considered the severity of insomnia, and the ORs (95% CIs) of multimorbidity were 2.0 (1.8–2.2), 2.0 (1.8–2.1) and 2.6 (2.3 = 3.0) for the moderate insomnia, insomnia with fatigue and insomnia diagnosis, respectively.

### Snoring and Multimorbidity

Three cross-sectional studies on snoring were not entered the meta-analyses, all of which showed significant association with multimorbidity [28, 40, 43]. Appleton et al. [28] recruited 1,011 Australian adults and observed an OR of 2.4 (95% CI = 1.2–4.5) for multimorbidity (2 or more vs. 0). Torsza et al. [40] based on 12,643 Hungarian suggested the positive association of snoring with 3 or more comorbid conditions (OR = 1.46, 95% CI = 1.31–1.63). Another cross-sectional study of 12,765 adults aged 35–74 years conducted by Zhang et al. [43] found that “snoring sometimes” presented an OR of 1.61 for multimorbidity in males (95% 1.40–1.86), and of 2.03 in females (1.82–2.27), while “snoring more frequently” presented an OR of 1.75 for multimorbidity in males (95% CI = 1.39–2.21), and of 1.97 in females (95% CI = 1.59–2.44). The definition of snoring and main results were summarized in Table 2.

**TABLE 2:** The definition of snoring, poor sleep quality, obstructive sleep apnea and restless legs syndrome and results of their association with multimorbidity (Australia, Canada, China, Germany, Hungary and Italy. 2011–2020).

Study	The definition of sleep problems	Results
Snoring		
Appleton et al. [28]	Loud snoring ≥3 times per week without witnessed breathing pauses	Having ≥2 physician-diagnosed medical conditions was associated with simple snoring (OR [95% CI]: 2.3 [1.2–4.4])
Torzsa et al. [40]	Self-report of habitual snoring or loud snoring with breathing pauses	The presence of three or more co-morbid conditions was independent predictors of snoring (OR [95% CI]: 1.45 [1.30–1.62])
Zhang et al. [43]	Self-report of snoring	The subjects with snoring frequently (OR = 1.88, 95% CI = 1.61–2.21) had a higher risk of chronic comorbidities
Poor sleep quality		
Liu et al. [33]	Self-report of having sleep conditions	Sleep condition was the influencing factors of chronic disease comorbidities
Nicholson et al. [34]	Self-report of being dissatisfied with current sleep pattern	The odds of multimorbidity were higher for participants who self-reported dissatisfaction with sleep quality
OSA		
Appleton et al. [28]	Diagnosed OSA: Self-report of having been diagnosed with sleep apnea with an overnight sleep study Undiagnosed OSA: (1) witnessed breathing pauses ≥3 times per week or (2) witnessed breathing pauses ≥3 times per month with loud snoring ≥3 times per week	Having ≥2 physician-diagnosed medical conditions was associated with diagnosed OSA (OR [95% CI]: 8.8 [4.1–18.7]), undiagnosed OSA (2.9 [1.6–5.3])
Lacedonia et al. [31]	No obstructive pulmonary disease, PaCO_2_ below 45 mmHg	The presence and the association of ≥3 comorbidities seem to be higher in patients suffering from OSA, but the effect size was not significant (OR = 1.36, 95% CI = 0.75–2.45)
Robichaud-Hallé et al. [35]	Based on an AHI value (absent: AHI 0–4; mild: AHI 5–14; moderate: AHI 15–29; severe: AHI ≥30)	Severe OSA (AHI≥30) was associated with Median DBMA (OR = 3.94, 95% CI = 1.24–12.59), DBMA 10 (OR = 4.34, 95% CI = 1.22–15.44) and DBMA 20 (OR = 7.33, 95% CI = 1.67–32.23)
Ruel et al. [36]	Based on an AHI value (absent/none: AHI<10; mild: AHI ≥10 and <20; moderate: AHI≥20 and <30; severe: AHI≥30)	Multimorbidity was associated with AHI and undiagnosed OSA.
RLS		
Appleton et al. [28]	Unpleasant, tingling, or restless feelings in the legs at least a few times per month	Having ≥2 physician-diagnosed medical conditions was associated with restless legs (OR [95% CI]: 1.9 [1.2–3.1])
Szentkirályi et al. [39]	Self-report of having all symptoms of RLS	An increase in the number of comorbid conditions at baseline predicted prevalent RLS (DHS: trend OR = 1.24, 95% CI = 0.99–1.56; SHIP: trend OR = 1.34, 95% CI = 1.18–1.52) and incident RLS (DHS: trend OR = 1.32, 95% CI = 1.04–1.68; SHIP: trend OR = 1.59, 95% CI = 1.37–1.85) after adjustment for several covariates. The ORs for incident RLS associated with 3 or more comorbid diseases (DHS: OR = 2.51, 95% CI = 1.18–5.34; SHIP: OR = 4.30, 95% CI = 2.60–7.11) were higher than the ORs for any single disease

Abbreviations: OSA, obstructive sleep apnea; RLS, restless legs syndrome; AHI, apnea-hypopnea index; DBMA, disease burden morbidity assessment; OR, odds ratio; CI, confidence interval; DHS, the Dortmund Health Study; SHIP, the study of health in Pomerania.

### Poor Sleep Quality and Multimorbidity

Two cross-sectional studies reported inconsistent evidence on the association between poor sleep quality and multimorbidity [33, 34]. Participants were asked whether having sleep conditions or being dissatisfied with current sleep pattern. The sex-stratified model of Canadian residents by Nicholson et al. regarded neutral sleep quality as the reference, and indicated that dissatisfied sleep quality was significantly associated with higher odds of multimorbidity in males (OR = 1.20, 95% CI = 1.02–1.41) but not in females (OR = 1.14, 95% CI = 0.99–1.32). Meanwhile, satisfied sleep quality was significantly associated with multimorbidity in only females (males: OR = 0.88, 95% CI = 0.76–1.00; females: OR = 0.87, 95% CI = 0.76–0.99), compared to neutral sleep quality [34]. In another cross-sectional study of 3,327 Mongolian residents in China, poor sleep quality was found to be a risk factor for multimorbidity (OR = 1.52, 95% CI = 1.29–1.79) [33]. The definition of poor sleep quality and main results were summarized in Table 2.

### OSA and Multimorbidity

Four cross-sectional studies reported on the potential association between OSA and multimorbidity [28, 31, 35, 36]. A cross-sectional study on 1,011 Australian adults by Appleton et al. [28] suggested a moderate-to-strong association between OSA and multimorbidity (diagnosed OSA: OR = 8.8, 95% CI = 4.1–18.7; possible undiagnosed OSA: OR = 2.9, 95% CI = 1.6–5.3) (2 or more vs. 0). Lacedonia et al. [31] revealed the prevalence of comorbidities increased in patients with OSA. The remaining two cross-sectional studies divided the OSA severity into four categories: absent (apnea-hypopnea index [AHI] 0–4), mild (AHI 5–14), moderate (AHI 15–29), and severe (AHI ≥30). Robichaud-Hallé et al. [35] used the DBMA score to measure multimorbidity, and found that severe OSA (AHI ≥30) was significantly associated with both DBMA 10 (referring to two or more chronic conditions, OR = 4.34, 95% CI = 1.22–15.44) and DBMA 20 (referring to four or more chronic conditions, OR = 7.33, 95% CI = 1.67–32.23). Ruel et al. [36] reported all severity groups of OSA (mild, moderate and severe) were associated with multimorbidity, while the strongest effect appeared in those with severe OSA (OR = 4.53, 95% CI = 1.82–11.30 for 3 or more conditions vs. 0–2 conditions). The definition of OSA and main results were summarized in Table 2.

### RLS and Multimorbidity

Two studies (one cross-sectional study and one cohort study) reported significant association between RLS and multimorbidity [28, 39]. Appleton et al. [28] found a moderate association between RLS and multimorbidity (OR = 2.4, 95% CI = 1.5–3.9, 2 or more vs. 0). Szentkirályi et al. conducted second analyses of two population-based databases [the Dortmund Health Study (DHS) and the Study of Health in Pomerania (SHIP)], respectively, and reported both cross-sectional and longitudinal association between RLS and multimorbidity. This study suggested that as the number of comorbidities increased, the prevalence (DHS: OR for trend per 1 condition increase = 1.24, 95% CI = 0.99–1.56; SHIP: OR for trend per 1 condition increase = 1.34, 95% CI = 1.18–1.52) and incidence (DHS: OR for trend per 1 condition increase = 1.32, 95% CI = 1.04–1.68; SHIP: OR for trend per 1 condition increase = 1.59, 95% CI = 1.37–1.85) of RLS both became higher. The definition of RLS and main results were summarized in Table 2.

### Sleep Problems and Individual Chronic Conditions

We further meta-analyzed the association of abnormal sleep duration with individual chronic conditions. The most common chronic conditions used to construct multimorbidity were hypertension, diabetes and heart disease, according to four studies [16, 32, 41, 42]. Short sleep duration (OR = 1.34, 95% CI = 1.21–1.49, I^2^ = 0%) and long sleep duration (OR = 1.47, 95% CI = 1.09–1.98, I^2^ = 71%) were observed to be associated with heart diseases (Supplementary Table S9).

## Discussion

In this systematic review of 17 observational studies, we summarized evidence on the association between six sleep problems and multimorbidity. Results from meta-analyses showed abnormal sleep duration and insomnia were associated with higher odds of multimorbidity. Sleep problems including snoring, poor sleep quality, OSA and RLS were narratively described in our review due to limited number of comparable studies. However, current studies of the above-mentioned sleep problems all revealed significant association with multimorbidity, except for one cross-sectional study conducted by Nicolson et al. [34], which found the effect sizes of poor sleep quality differed between sexes. Our study evaluated current evidence on which and to what extent sleep problems were linked to multimorbidity from a multi-faceted perspective, and provides a basis for future observational and experimental studies which aim to clarify the nature of the interplay between sleep problems and multimorbidity.

Previous evidence has shown the significant associations of abnormal sleep duration with a series of health outcomes. A systematic review and meta-analysis of 108 cohort studies found short sleep duration was associated with mortality, diabetes, hypertension, cardiovascular disease, coronary heart disease and obesity [17]. Another systematic review and meta-analysis included 137 cohort studies and reported the significant association of long sleep duration with mortality, diabetes, cardiovascular disease, coronary heart disease and obesity [18]. Both self-reported short and long sleep duration was reported to relate to metabolic syndrome [44]. Another systematic review found abnormal sleep duration played a role in predicting cardiovascular outcomes [45], supported by [46]. Systematic reviews on insomnia mainly focused on its mental and cognitive outcomes, including mental disorders, cognitive performance and dementia [20, 21, 47]. Encouragingly, a systematic review of 64 observational studies suggested that chronic insomnia or insomnia accompanied with short sleep duration, was strongly associated with hypertension [48]. In our study, results of meta-analyses showed that short and long sleep duration and insomnia were associated with higher odds of multimorbidity, despite of high heterogeneity. Meta-regression analyses suggested the definition of long sleep duration contributed significantly to the heterogeneity, which was reasonable because common sleep duration varies among cultures and ethnicities. For the association between insomnia and multimorbidity, the publication year was the effective variable to heterogeneity, suggesting the pattern and reason for the target association might change over time. Considering the increasing prevalence of unhealthy sleep patterns and chronic conditions, our study included a wide range of chronic conditions, and adds evidence on the association between sleep problems and multiple chronic conditions. This study also supports the candidacy of sleep promotion as an important strategy for prevention and management of multimorbidity. According to previous studies, both short sleep duration and insomnia were potentially characterized by objective insufficient sleep, and their relationship with multimorbidity could be interpreted by similar biological plausibility. There is evidence that short sleep duration can disrupt cardio-metabolic, endocrine, immune and inflammatory pathways [34]. For instance, insufficient sleep might influence hormones, such as cortisol, insulin and leptin [49, 50]. Circadian disruption and autonomic nervous system changes can also occur among individuals suffering from sleep loss [37]. The inflammatory system might also play a role in linking insomnia with multimorbidity [51, 52]. Pain, mental and physical discomfort, as well as the medications or treatments used, may all have an impact on the accumulation of sleep problems in patients with multimorbidity [16, 32, 37]. Compared to short sleep duration and insomnia, the underlying mechanisms for the association between long sleep duration and multimorbidity remain unknown. However, it has been assumed that long sleep is commonly associated with sleep disturbances and may be the complication of certain chronic conditions and psychiatric disorders [34].

The associations of the other four sleep problems with multimorbidity reported in primary studies were incomparable, therefore no meta-analysis was conducted. This incomparability might be related to the varied classification and severity of sleep problems (e.g., the frequency of snoring and the severity of OSA). Existing systematic reviews suggested poor sleep quality were an important predictor of cardiometabolic diseases, such as hypertension, coronary artery disease and metabolic syndrome [22, 53–56]. In our review, sex differences were found in the association between poor sleep quality and multimorbidity. Poor sleep quality was often accompanied by inadequate sleep, and might have similar pathways with short sleep duration and insomnia to multimorbidity [57]. The effects of snoring and OSA on health outcomes have been widely systematically reviewed, ranging from physical chronic conditions (cardiovascular diseases, cancer and osteoporosis) and psychiatric diseases (e.g., depression) to cognitive function and dementia [58–63]. In our review, the significant association between snoring and multimorbidity was reported among all three cross-sectional studies, regardless of snoring frequency. Three studies reported the significant association between OSA and multimorbidity, while one study find only those with severe OSA (AHI ≥30) experienced higher odds of multimorbidity. Given OSA has become increasingly common [64], the diagnosis and classification of OSA should be clarified in future observational and experimental studies. Snoring has been observed to be a major symptom of OSA [53, 65], and mechanical damage to the endothelial wall, inflammatory cascade and disorder of the neuroendocrine system were possible explanations proposed for the association of snoring and OSA with multimorbidity [54]. Moreover, accumulating evidence found these sleep problems did not affect individually. For example, poor sleep quality caused by snoring would also increase the excitability of the sympathetic nervous system and disrupt circadian rhythmicity [66]. Several systematic reviews have summarized the association between RLS and chronic conditions, with inconsistent results [67, 68]. The two studies included in our review both found the significant association between RLS and multimorbidity. The underlying mechanisms between RLS and multimorbidity remain unclear, and the knowledge gaps need to be filled. Sleep problems often co-exist with each other, making the underlying associations between sleep problems and multimorbidity to be complex and intermediary. Future research is warranted to gain a comprehensive understanding of sleep problems and its physiological characteristics. As current evidence regarding the mechanisms of sleep problems and multimorbidity was limited, more longitudinal studies are needed to confirm their underlying associations.

### Limitations of the Study

Limitations to this study warrant consideration. First, most of the included studies were cross-sectional in design, limiting our exploration of the longitudinal association between sleep problems and multimorbidity. Second, data on sleep and multimorbidity was self-reported, which was subject to recall bias. However, previous studies have found a moderate correlation between objective and subjective measurement [34, 69]. In addition, the inconsistent definition of sleep problems and multimorbidity led to the high heterogeneity of our study, and limited our ability to summarize conclusive evidence. Although many common conditions like hypertension, diabetes and heart diseases were considered in most studies, standard definition and measurement of multimorbidity can still be a priority [13]. Third, according to the included studies, multimorbidity was based on a restricted list of chronic conditions, which would cause underestimation on the prevalence of multimorbidity. This limitation also precludes us to further meta-analyze the association between sleep problems and single chronic conditions. Fourth, according to previous evidence, the prevalence of sleep problems and multimorbidity both increased by age, but exploration of the role of age on the association between sleep problems and multimorbidity was not allowed due to the limited information of population age from primary studies. Finally, many primary studies failed to distinguish the definition of multimorbidity and comorbidity and used them interchangeably, which would potentially lead to the misjudgment during study selection. Studies with cohort in design, larger sample sizes and clear definition of variables are needed in the future to capture the true association between sleep problems and multimorbidity.

## Conclusion

Of the six sleep problems included in our systematic review, abnormal sleep duration and insomnia were associated with multimorbidity, while the association of snoring, poor sleep quality, OSA and RLS with multimorbidity remains inconclusive. Large prospective studies with long-term follow-up on the association between sleep problems and multimorbidity are warranted. Interventions targeting sleep problems may have the potential to support better management of multimorbidity.

## Abbreviations

AHRQ, the Agency for Healthcare Research and Quality; AHI, apnea-hypopnea index; CI, confidence interval; CVD, cardiovascular disease; DBMA, the disease burden morbidity assessment; DHS, the Dortmund Health Study; NOS, the Newcastle-Ottawa scale; OR, odd ratio; OSA, obstructive sleep apnea; RLS, restless legs syndrome; SHIP, the study of health in Pomerania.
