# Supplementary material for: Sleep Problems Associate With Multimorbidity: A Systematic Review and Meta-analysis
Source: Public Health Rev. 2023 Jun 13;44:1605469. doi: 10.3389/phrs.2023.1605469 (PMC10293634; doi:10.3389/phrs.2023.1605469)
Supplement: Supplementary file 1 [file DataSheet1.docx]

**Supplemental Material**

**Table S1** PubMed search strategy

**Table S2** Web of science search strategy

**Table S3** Embase search strategy

**Table S4** Quality Assessment Tools for cross-sectional and cohort studies.

**Table S5** Excluded studies and reasons.

**Table S6** Detailed characteristics of included studies.

**Table S7** The quality assessment for cross-sectional studies.

**Table S8** The quality assessment for cohort studies.s

**Table S9** Associations between abnormal sleep duration and some commonly included chronic conditions in the definition of multimorbidity.

**Figure S1** Meta-analysis of the association between sleep duration and multimorbidity from cross-sectional studies. (A) Short sleep duration and multimorbidity, stratified by the definition of multimorbidity; (B) long sleep duration and multimorbidity, stratified by the definition of long sleep duration.

Normal sleep duration: Helbig et al. (2017) –7-8h; Nicholson et al. (2020) –6-8h; Reis et al. (2018) –6-8h; Zhang et al. (2019) – 8h; Wang et al. (2017) a –7-9h; Wang et al. (2017) b –7-8h; Lima et al. (2021) – 7-8h; Ruiz-Castell et al. (2019) – 6-9h.

**Figure S2** Meta-analysis of the association between short sleep duration and multimorbidity after removing three outliers.

**Figure S3** Funnel plot of studies on abnormal sleep duration and multimorbidity, with the odds ratio on the horizontal axis, and the standard error on the vertical axis. (A) short sleep duration; (B) long sleep duration.

**Figure S4** Meta-analysis of the association between insomnia and multimorbidity from cross-sectional studies, stratified by publication years.

**Figure S5** Meta-analysis of the association between insomnia and multimorbidity after removing one outlier.

**Figure S6** Funnel plot of studies on insomnia and multimorbidity, with the odds ratio on the horizontal axis, and the standard error on the vertical axis.

**Table S1** PubMed search strategy.

| No. | Query | Results |
| --- | --- | --- |
| #1 | "associated condition*"[Title/Abstract] OR "associated diagnos*"[Title/Abstract] OR "associated disease*"[Title/Abstract] OR "associated health problem*"[Title/Abstract] OR "associated illness*"[Title/Abstract] OR "associated morbidity"[Title/Abstract] OR "associated morbidities"[Title/Abstract] OR "associated pathology"[Title/Abstract] OR "associated pathologies"[Title/Abstract] | 29891 |
| #2 | "coexisting condition*"[Title/Abstract] OR "co-existing condition*"[Title/Abstract] OR "coexisting diagnos*"[Title/Abstract] OR "co-existing diagnos*"[Title/Abstract] OR "coexisting disease*"[Title/Abstract] OR "co-existing disease*"[Title/Abstract] OR "coexisting health problem*"[Title/Abstract] OR "co-existing health problem*"[Title/Abstract] OR "coexisting illness*"[Title/Abstract] OR "co-existing illness*"[Title/Abstract] OR "coexisting morbid*"[Title/Abstract] OR "co-existing morbid*"[Title/Abstract] OR "coexisting patholog*"[Title/Abstract] OR "co-existing patholog*"[Title/Abstract] | 2510 |
| #3 | "comorbid condition*"[Title/Abstract] OR "co-morbid condition*"[Title/Abstract] OR "comorbid diagnos*"[Title/Abstract] OR "co-morbid diagnos*"[Title/Abstract] OR "comorbid disease*"[Title/Abstract] OR "co-morbid disease*"[Title/Abstract] OR "comorbid health problem*"[Title/Abstract] OR "co-morbid health problem*"[Title/Abstract] OR "comorbid illness*"[Title/Abstract] OR "co-morbid illness*"[Title/Abstract] OR "comorbid patholog*"[Title/Abstract] OR "co-morbid patholog*"[Title/Abstract] | 21132 |
| #4 | "concurrent condition"[Title/Abstract] OR "concurrent conditions"[Title/Abstract] OR "concurrent diagnos*"[Title/Abstract] OR "concurrent disease*"[Title/Abstract] OR "concurrent health problem*"[Title/Abstract] OR "concurrent illness*"[Title/Abstract] OR "concurrent morbid*"[Title/Abstract] OR "concurrent patholog*"[Title/Abstract] OR "cooccurring condition*"[Title/Abstract] OR "co-occurring condition*"[Title/Abstract] OR "cooccurring diagnos*"[Title/Abstract] OR "co-occurring diagnos*"[Title/Abstract] OR "cooccurring disease*"[Title/Abstract] OR "co-occurring disease*"[Title/Abstract] OR "cooccurring health problem*"[Title/Abstract] OR "co-occurring health problem*"[Title/Abstract] OR "cooccurring illness*"[Title/Abstract] OR "co-occurring illness*"[Title/Abstract] OR "cooccurring morbid*"[Title/Abstract] OR "co-occurring morbid*"[Title/Abstract] OR "cooccurring patholog*"[Title/Abstract] OR "co-occurring patholog*"[Title/Abstract] | 3087 |
| #5 | "multiple condition"[Title/Abstract] OR "multiple conditions"[Title/Abstract] OR "multiple diagnosis"[Title/Abstract] OR "multiple diagnoses"[Title/Abstract] OR "multiple disease"[Title/Abstract] OR "multiple diseases"[Title/Abstract] OR "multiple health problem*"[Title/Abstract] OR "multiple illness*"[Title/Abstract] OR "multiple morbid*"[Title/Abstract] OR "multiple pathology"[Title/Abstract] OR "multiple pathologies"[Title/Abstract] | 7024 |
| #6 | "multiple chronic disease"[Title/Abstract] OR "multiple chronic diseases"[Title/Abstract] OR "multiple chronic condition"[Title/Abstract] OR "multiple chronic conditions"[Title/Abstract] | 1986 |
| #7 | Comorbidity [MeSH Major Topic] | 4452 |
| #8 | "comorbidity" [Title/Abstract] OR "comorbidities"[Title/Abstract] OR "co-morbidity"[Title/Abstract] OR "co-morbidities"[Title/Abstract] | 177076 |
| #9 | multidisease[Title/Abstract] OR multi-disease[Title/Abstract] OR multi-diseases[Title/Abstract] OR multimorbidity[Title/Abstract] OR multimorbidities[Title/Abstract] OR multi-morbidity[Title/Abstract] OR multi-morbidities[Title/Abstract] OR multipathology[Title/Abstract] OR multipathologies[Title/Abstract] OR multi-pathology[Title/Abstract] OR multi-pathologies[Title/Abstract] | 6803 |
| #10 | pluripathology OR pluripathologies OR polypathology OR polypathologies OR poly-pathology OR poly-pathologies | 162 |
| #11 | pluripathology[Title/Abstract] OR pluripathologies[Title/Abstract] OR polypathology[Title/Abstract] OR polypathologies[Title/Abstract] OR poly-pathology[Title/Abstract] OR poly-pathologies[Title/Abstract] | 149 |
| #12 | #1 OR #2 OR #3 OR #4 OR #5 OR #6 OR #7 OR #8 OR #9 OR #10 OR #11 | 239583 |
| #13 | humans[Filter] AND English[Language] | 16631292 |
| #14 | sleep[Title/Abstract] OR sleep disorder[Title/Abstract] OR sleep problems[Title/Abstract] OR sleep disturbances[Title/Abstract] OR sleep quality[Title/Abstract] OR insomnia[Title/Abstract] OR sleepiness[Title/Abstract] OR somnolence[Title/Abstract] OR sleep apnea[Title/Abstract] OR snoring[Title/Abstract] OR sleep disordered breathing[Title/Abstract] OR obstructive sleep apnea[Title/Abstract] OR restless legs syndrome[Title/Abstract] OR periodic limb movements disorder[Title/Abstract] OR REM sleep behavior disorder[Title/Abstract] OR sleep movement disorder[Title/Abstract] OR parasomnias[Title/Abstract] OR sleep-wake disruption[Title/Abstract] OR circadian rhythm sleep disorder[Title/Abstract] OR sleep phase[Title/Abstract] OR sleep pattern[Title/Abstract] OR sleep duration[Title/Abstract] OR nap[Title/Abstract] OR napping | 212417 |
| #15 | #12 AND #13 AND #14 | 6858 |

**Table S2** Web of science search strategy.

| No. | Query | Results |
| --- | --- | --- |
| #1 | (AB=("associated condition*" OR "associated diagnos*" OR "associated disease*" OR "associated health problem*" OR "associated illness*" OR “associated morbidity” OR “associated morbidities” OR “associated pathology” OR “associated pathologies”) ) AND LANGUAGE:(English) | 24121 |
| #2 | (TI=("associated condition*" OR "associated diagnos*" OR "associated disease*" OR "associated health problem*" OR "associated illness*" OR "associated morbidity" OR "associated morbidities" OR "associated pathology" OR "associated pathologies") ) AND LANGUAGE:(English) | 3289 |
| #3 | (AB=(“coexisting condition*” OR “co-existing condition*” OR “coexisting diagnos*” OR “co-existing diagnos*” OR “coexisting disease*” OR “co-existing disease*” OR “coexisting health problem*” OR “co-existing health problem*” OR “coexisting illness*” OR “co-existing illness*” OR “coexisting morbid*” OR “co-existing morbid*” OR “coexisting patholog*” OR “co-existing patholog*”) ) AND LANGUAGE:(English) | 1942 |
| #4 | (TI=(“coexisting condition*” OR “co-existing condition*” OR “coexisting diagnos*” OR “co-existing diagnos*” OR “coexisting disease*” OR “co-existing disease*” OR “coexisting health problem*” OR “co-existing health problem*” OR “coexisting illness*” OR “co-existing illness*” OR “coexisting morbid*” OR “co-existing morbid*” OR “coexisting patholog*” OR “co-existing patholog*”) ) AND LANGUAGE: (English) | 132 |
| #5 | (AB=("comorbid condition*" OR "co-morbid condition*" OR "comorbid diagnos*" OR "co-morbid diagnos*" OR "comorbid disease*" OR "co-morbid disease*" OR "comorbid health problem*" OR "co-morbid health problem*" OR "comorbid illness*" OR "co-morbid illness*" OR "comorbid patholog*" OR "co-morbid patholog*") ) AND LANGUAGE: (English) | 18662 |
| #6 | (TI=("comorbid condition*" OR "co-morbid condition*" OR "comorbid diagnos*" OR "co-morbid diagnos*" OR "comorbid disease*" OR "co-morbid disease*" OR "comorbid health problem*" OR "co-morbid health problem*" OR "comorbid illness*" OR "co-morbid illness*" OR "comorbid patholog*" OR "co-morbid patholog*") ) AND LANGUAGE: (English) | 1358 |
| #7 | (AB=(“concurrent condition” OR “concurrent conditions” OR “concurrent diagnos*” OR “concurrent disease*” OR “concurrent health problem*” OR “concurrent illness*” OR “concurrent morbid*” OR “concurrent patholog*” OR “cooccurring condition*” OR “co-occurring condition*” OR “cooccurring diagnos*” OR “co-occurring diagnos*” OR “cooccurring disease*” OR “co-occurring disease*” OR “cooccurring health problem*” OR “co-occurring health problem*” OR “cooccurring illness*” OR “co-occurring illness*” OR “cooccurring morbid*” OR “co-occurring morbid*” OR “cooccurring patholog*” OR “co-occurring patholog*”) ) AND LANGUAGE: (English) | 2545 |
| #8 | (TI=(“concurrent condition” OR “concurrent conditions” OR “concurrent diagnos*” OR “concurrent disease*” OR “concurrent health problem*” OR “concurrent illness*” OR “concurrent morbid*” OR “concurrent patholog*” OR “cooccurring condition*” OR “co-occurring condition*” OR “cooccurring diagnos*” OR “co-occurring diagnos*” OR “cooccurring disease*” OR “co-occurring disease*” OR “cooccurring health problem*” OR “co-occurring health problem*” OR “cooccurring illness*” OR “co-occurring illness*” OR “cooccurring morbid*” OR “co-occurring morbid*” OR “cooccurring patholog*” OR “co-occurring patholog*”) ) AND LANGUAGE: (English) | 201 |
| #9 | (AB=(“multiple condition” OR “multiple conditions” OR “multiple diagnosis” OR “multiple diagnoses” OR “multiple disease” OR “multiple diseases” OR “multiple health problem*” OR “multiple illness*” OR “multiple morbid*” OR “multiple pathology” OR “multiple pathologies”) ) AND LANGUAGE: (English) | 6705 |
| #10 | (TI=("multiple condition" OR "multiple conditions" OR "multiple diagnosis" OR "multiple diagnoses" OR "multiple disease" OR "multiple diseases" OR "multiple health problem*" OR "multiple illness*" OR "multiple morbid*" OR "multiple pathology" OR "multiple pathologies") ) AND LANGUAGE: (English) | 741 |
| #11 | (AB=(“multiple chronic disease” OR “multiple chronic diseases” OR “multiple chronic condition” OR “multiple chronic conditions”)  ) AND LANGUAGE: (English) | 1535 |
| #12 | (TI=(“multiple chronic disease” OR “multiple chronic diseases” OR “multiple chronic condition” OR “multiple chronic conditions”) ) AND LANGUAGE: (English) | 601 |
| #13 | (TS=Comorbidity) AND LANGUAGE: (English) | 99176 |
| #14 | (AB=(“comorbidity” OR “comorbidities” OR “co-morbidity” OR “co-morbidities”) ) AND LANGUAGE: (English) | 156004 |
| #15 | (TI=(“comorbidity” OR “comorbidities” OR “co-morbidity” OR “co-morbidities”) ) AND LANGUAGE: (English) | 26541 |
| #16 | (AB=(multidisease OR multi-disease OR multi-diseases OR multimorbidity OR multimorbidities OR multi-morbidity OR multi-morbidities OR multipathology OR multipathologies OR multi-pathology OR multi-pathologies) ) AND LANGUAGE: (English) | 5249 |
| #17 | (TI==(multidisease OR multi-disease OR multi-diseases OR multimorbidity OR multimorbidities OR multi-morbidity OR multi-morbidities OR multipathology OR multipathologies OR multi-pathology OR multi-pathologies) ) AND LANGUAGE: (English) | 3221 |
| #18 | (ALL=(pluripathology OR pluripathologies OR polypathology OR polypathologies OR poly-pathology OR poly-pathologies) ) AND LANGUAGE: (English) | 149 |
| #19 | (AB=(pluripathology OR pluripathologies OR polypathology OR polypathologies OR poly-pathology OR poly-pathologies) ) AND LANGUAGE: (English) | 100 |
| #20 | (TI=(pluripathology OR pluripathologies OR polypathology OR polypathologies OR poly-pathology OR poly-pathologies) ) AND LANGUAGE: (English) | 36 |
| #21 | #20 OR #19 OR #18 OR #17 OR #16 OR #15 OR #14 OR #13 OR #12 OR #11 OR #10 OR #9 OR #8 OR #7 OR #6 OR #5 OR #4 OR #3 OR #2 OR #1 | 255377 |
| #22 | (AB=(sleep OR sleep disorder OR sleep problems OR sleep disturbances OR sleep quality OR insomnia OR sleepiness OR somnolence OR sleep apnea OR snoring OR sleep disordered breathing OR obstructive sleep apnea OR restless legs syndrome OR periodic limb movements disorder OR REM sleep behavior disorder OR sleep movement disorder OR parasomnias OR sleep-wake disruption OR circadian rhythm sleep disorder OR sleep phase OR sleep pattern OR sleep duration OR nap OR napping) ) *AND***LANGUAGE:** (English) | 188246 |
| #23 | (TI=(sleep OR sleep disorder OR sleep problems OR sleep disturbances OR sleep quality OR insomnia OR sleepiness OR somnolence OR sleep apnea OR snoring OR sleep disordered breathing OR obstructive sleep apnea OR restless legs syndrome OR periodic limb movements disorder OR REM sleep behavior disorder OR sleep movement disorder OR parasomnias OR sleep-wake disruption OR circadian rhythm sleep disorder OR sleep phase OR sleep pattern OR sleep duration OR nap OR napping) ) *AND***LANGUAGE:** (English) | 170139 |
| #24 | #23 OR #22 | 263757 |
| #25 | #24 AND #21 | 8707 |

**Table S3** Embase search strategy.

| No. | Query | Results |
| --- | --- | --- |
| #1 | 'associated condition*':ab,ti OR 'associated diagnos*':ab,ti OR 'associated disease*':ab,ti OR 'associated health problem*':ab,ti OR 'associated illness*':ab,ti OR 'associated morbidity':ab,ti OR 'associated morbidities':ab,ti OR 'associated pathology':ab,ti OR 'associated pathologies':ab,ti | 40032 |
| #2 | 'comorbid condition*':ab,ti OR 'co-morbid condition*':ab,ti OR 'comorbid diagnos*':ab,ti OR 'co-morbid diagnos*':ab,ti OR 'comorbid disease*':ab,ti OR 'co-morbid disease*':ab,ti OR 'comorbid health problem*':ab,ti OR 'co-morbid health problem*':ab,ti OR 'comorbid illness*':ab,ti OR 'co-morbid illness*':ab,ti OR 'comorbid patholog*':ab,ti OR 'co-morbid patholog*':ab,ti | 34766 |
| #3 | 'coexisting condition*':ab,ti OR 'co-existing condition*':ab,ti OR 'coexisting diagnos*':ab,ti OR 'co-existing diagnos*':ab,ti OR 'coexisting disease*':ab,ti OR 'co-existing disease*':ab,ti OR 'coexisting health problem*':ab,ti OR 'co-existing health problem*':ab,ti OR 'coexisting illness*':ab,ti OR 'co-existing illness*':ab,ti OR 'coexisting m bid*':ab,ti OR 'co-existing m bid*':ab,ti OR 'coexisting patholog*':ab,ti OR 'co-existing patholog*':ab,ti | 3640 |
| #4 | 'concurrent condition':ab,ti OR 'concurrent conditions':ab,ti OR 'concurrent diagnos*':ab,ti OR 'concurrent disease*':ab,ti OR 'concurrent health problem*':ab,ti OR 'concurrent illness*':ab,ti OR 'concurrent morbid*':ab,ti OR 'concurrent patholog*':ab,ti OR 'cooccurring condition*':ab,ti OR 'co-occurring condition*':ab,ti OR 'cooccurring diagnos*':ab,ti OR 'co-occurring diagnos*':ab,ti OR 'cooccurring disease*':ab,ti OR 'co-occurring disease*':ab,ti OR 'cooccurring health problem*':ab,ti OR 'co-occurring health problem*':ab,ti OR 'cooccurring illness*':ab,ti OR 'co-occurring illness*':ab,ti OR 'cooccurring morbid*':ab,ti OR 'co-occurring morbid*':ab,ti OR 'cooccurring patholog*':ab,ti OR 'co-occurring patholog*':ab,ti | 4664 |
| #5 | 'multiple chronic disease':ab,ti OR 'multiple chronic diseases':ab,ti OR 'multiple chronic condition':ab,ti OR 'multiple chronic conditions':ab,ti | 2242 |
| #6 | 'comorbidity'/mj | 19077 |
| #7 | 'multiple condition':ab,ti OR 'multiple conditions':ab,ti OR 'multiple diagnosis':ab,ti OR 'multiple diagnoses':ab,ti OR 'multiple disease':ab,ti OR 'multiple diseases':ab,ti OR 'multiple health problem*':ab,ti OR 'multiple illness*':ab,ti OR 'multiple morbid*':ab,ti OR 'multiple pathology':ab,ti OR 'multiple pathologies':ab,ti | 9302 |
| #8 | multidisease:ab,ti OR 'multi disease':ab,ti OR 'multi diseases':ab,ti OR multimorbidity:ab,ti OR multimorbidities:ab,ti OR 'multi morbidity':ab,ti OR 'multi morbidities':ab,ti OR multipathology:ab,ti OR multipathologies:ab,ti OR 'multi pathology':ab,ti OR 'multi pathologies':ab,ti OR pluripathology:ab,ti | 7752 |
| #9 | pluripathology:ab,ti OR pluripathologies:ab,ti OR polypathology:ab,ti OR polypathologies:ab,ti OR 'poly pathology':ab,ti OR 'poly pathologies':ab,ti | 287 |
| #10 | sleep:ab,ti OR 'sleep disorder':ab,ti OR 'sleep problems':ab,ti OR 'sleep problem':ab,ti OR 'sleep disturbances':ab,ti OR 'sleep disturbance':ab,ti OR 'sleep quality':ab,ti OR insomnia:ab,ti OR sleepiness:ab,ti OR somnolence:ab,ti OR 'sleep apnea':ab,ti OR snoring:ab,ti OR 'sleep disordered breathing':ab,ti OR 'obstructive sleep apnea':ab,ti OR 'restless legs syndrome':ab,ti OR 'periodic limb movements disorder':ab,ti OR 'rem sleep behavior disorder':ab,ti OR 'sleep movement disorder':ab,ti OR parasomnias:ab,ti OR 'sleep-wake disruption':ab,ti OR 'circadian rhythm sleep disorder':ab,ti OR 'sleep phase':ab,ti OR 'sleep pattern':ab,ti OR 'sleep duration':ab,ti OR nap:ab,ti OR napping:ab,ti | 317747 |
| #11 | [humans]/lim | 23745893 |
| #12 | english:la | 33552727 |
| #13 | #1 OR #2 OR #3 OR #4 OR #5 OR #6 OR #7 OR #8 OR #9 | 116546 |
| #14 | #10 AND #13 | 4282 |
| #15 | #11 AND #12 AND #14 | 3885 |

**Table S4** Quality Assessment Tools for cross-sectional and cohort studies.

| Quality Assessment Tools | Categories | Item |
| --- | --- | --- |
| Methodology checklist by AHRQ for cross-sectional studies | / | 1) Define the source of information (survey, record review) |
|  |  | 2) List inclusion and exclusion criteria for exposed and unexposed subjects (cases and controls) or refer to previous publications |
|  |  | 3) Indicate time period used for identifying patients |
|  |  | 4) Indicate whether or not subjects were consecutive if not population-based |
|  |  | 5) Indicate if evaluators of subjective components of study were masked to other aspects of the status of the participants |
|  |  | 6) Describe any assessments undertaken for quality assurance purposes (e.g., test/retest of primary outcome measurements) |
|  |  | 7) Explain any patient exclusions from analysis |
|  |  | 8) Describe how confounding was assessed and/or controlled. |
|  |  | 9) If applicable, explain how missing data were handled in the analysis |
|  |  | 10) Summarize patient response rates and completeness of data collection |
|  |  | 11) Clarify what follow-up, if any, was expected and the percentage of patients for which incomplete data or follow-up was obtained |
| NOS for cohort studies | Selection | Representativeness of the exposed cohort |
|  |  | Selection of the non-exposed cohort |
|  |  | Ascertainment of exposure |
|  |  | Demonstration that outcome of interest was not present at start of study |
|  | Comparability | Comparability of cohorts on the basis of the design or analysis |
|  | Outcome | Assessment of outcome |
|  |  | Was follow-up long enough for outcomes to occur |
|  |  | Adequacy of follow up of cohorts |

**Abbreviations:** AHRQ, Agency for Healthcare Research and Quality; NOS, Newcastle-Ottawa Scale.

**Table S5** Excluded studies and reasons.

| **Reasons** | **Number** |
| --- | --- |
| **Topics** | **387** |
| Not sleep problems | 18 |
| Not multimorbidity |  |
| Comorbidity |  |
| Lung diseases | 194 |
| Heart diseases | 83 |
| Single chronic conditions | 72 |
| Others | 20 |
| **Study object** | **68** |
| Animals | 37 |
| Only children or adolescents | 31 |
| **Results presentation** | **30** |
| No results for multimorbidity | 18 |
| Unknown sleep problems | 7 |
| No comparable statistical indicators | 5 |
| **Study design** | **93** |
| Review | 47 |
| Conference Abstract | 34 |
| Case reports | 6 |
| Clinical trial | 1 |
| Editorial | 2 |
| Letter | 3 |
| **Language** | **1** |
| Not English or Chinese | 1 |

**Table S6** Detailed characteristics of included studies.

| Study | Age range (Mean age) | Sample size | Male (%) | Classification of sleep problems | Included chronic diseases | Adjusted covariates |
| --- | --- | --- | --- | --- | --- | --- |
| Appleton et al. (2018) | ≥18 (NA) | 1011 | 49.8 | Diagnosed OSA: yes, no  Undiagnosed OSA: yes, no  Insomnia: yes, no  RLS: yes, no  Snoring: yes, no | Heart disease, diabetes, hypertension, reflux disease, lung disease, depression, anxiety/panic disorder, arthritis, cardiometabolic condition, mental health disorder | Sex, age, BMI, Epworth Sleepiness Scale, smoking |
| He et al. (2021) | ≥45 (NA) | 5321 | 45.3 | Sleep duration: poor (<7h or >9h), good (7-9h) | Heart disease, stroke, cancer, chronic lung disease, digestive disease, liver disease, kidney disease, memory-related disease [such as dementia, brain atrophy and/or Parkinson’s disease], arthritis and asthma | Survey year, age, educational level, marital status, living place, household income, health insurance, Body Mass Index (BMI), smoking cigarettes, drinking alcohol, depression and physical activity |
| Helbig et al. (2017) | 65-93 (73.0) | 3833 | 48.7 | Insomnia: yes, no  Sleep duration: short (≤5h), long (≥10h)  Trouble falling sleep: yes, no  Difficulty staying sleep: yes, no  Daytime tiredness: yes, no | Asthma, emphysema or chronic bronchitis, arthritis or rheumatism, cancer, diabetes, digestive problems, heart trouble, kidney disease, liver problems, stroke, HIV illness or AIDS, depression/anxiety, neurological diseases, eye diseases and hypertension | Age, sex, BMI, education status, risk of malnutrition, physical activity, frequent alcohol consumption, currently smoking and polypharmacy |
| Lacedonia et al. (2018) | NA (59.5) | 989 | 72.8 | OSA: yes, no | Arterial hypertension, heart disease (arrhythmias, heart failure, history of ischemic diseases, etc.), endocrinopathies (diabetes mellitus and thyroid disorders in particular), and metabolic syndrome | NA |
| Lima et al. (2012) | >18 (41.8) | 2637 | 47.7 | Sleep duration: short (<6h), normal (7-8h), long (>9h) | Hypertension, diabetes, heart disease, cancer, rheumatism/arthritis/arthrosis, osteoporosis, respiratory problems, repetitive strain injury, cardiovascular problems, emotional problem/anxiety/depression, migraine/headache, back problems, allergy, and vertigo | Gender, age, marital status, schooling, and work status |
| Liu et al. (2020) | ≥45 (NA) | 3327 | 39.3 | Sleep quality: good, poor | Hypertension, diabetes, dyslipidemia, rheumatic and rheumatoid diseases, coronary heart disease, chronic gastritis, intervertebral disc herniation, chronic gastritis, stroke, chronic nephritis and malignant neoplasms | Age, gender, education, occupation, annual household income, smoking, drinking, diet, farming and animal husbandry, physical examination frequency, file management, follow-up, chronic disease expenditure, mental health |
| Nicholson et al. (2020) | 45-85 (59.5) | 30011 | NA | Sleep duration: short (<6h), normal (6-8h), long (>8h)  Sleep quality: dissatisfied or very dissatisfied, neutral (reference), satisfied or very satisfied | Alzheimer’s disease, anxiety or mood disorder, arthritis, asthma, cancer, chronic obstructive pulmonary disease, diabetes, heart disease and stroke | Age group, ethnicity, education level, smoking status, alcohol consumption, activity level, sleep apnea, insomnia, BMI and hypertension |
| Reis et al. (2018) | ≥18 (56.7) | 5436 | 36.1 | Sleep duration: short (<5h), normal (5-9h), long (>9h) | Hypercholesterolemia, hypertension, rheumatic disease, allergy, gastrointestinal disease, cardiac disease, diabetes, pulmonary disease, cancer, or neurologic disease, depression and anxiety | Age, sex, Nomenclature of Territorial Units for Statistics and educational level |
| Robichaud-Hallé et al. (2012) | 30-75 (55.5) | 120 | NA | OSA: absent (AHI 0-4), mild (AHI 5-14), moderate (AHI 15-29), severe (AHI≥ 30) | Hypertension, heart disease, dyslipidemia, heart failure and stroke, cholesterol, obesity and diabetes | Sex, age, BMI and income |
| Ruel et al. (2018) | 41-87 (59.0) | 743 | 100 | Undiagnosed OSA: absent/none (AHI<10), mild (AHI≥10 and <20), moderate (AHI≥20 and <30), and severe (AHI≥30) | Asthma, heart disease, diabetes, depression, hyperlipidemia, hypertension, obesity, osteoarthritis and rheumatoid arthritis | Age, gender, marital status  and education level, household income, receiving government benefits or pension and employment status |
| Ruiz-Castell et al. (2019) | 25-64 (NA) | 1508 | 47.6 | Sleep duration: short (<6h), normal (6-9h), long (>9h). | Hypertension, high cholesterol, diabetes, cardiovascular diseases, stomach or duodenal ulcer, cirrhosis or other liver disease, urinary incontinence, kidney problems, chronic back or neck disorder, rheumatoid arthritis, arthrosis, osteoporosis, cancer, severe headache as migraine or chronic anxiety | Sociodemographic characteristics, behavioral risk factors and measures |
| Stewart et al. (2006) | 16-74 (NA) | 8580 | 44.9 | Insomnia: any, (at least) moderate severity, with fatigue, insomnia diagnosis | Longstanding health problems in each of 10 organ systems | NA |
| Szentkirályi et al. (2014) | 25-75 (50.7) | 5620 | 48.8 | RLS: yes, no | Diabetes, obesity, hypertension, cancer, myocardial infarction, stroke, kidney disease, anemia, thyroid disease, depressive symptoms and migraine | Age, sex, education, alcohol consumption, smoking, and physical activity |
| Torzsa et al. (2011) | NA (47.6) | 12643 | 43.2 | Snoring: non-snorers, habitual snorers, loud snorers | Cardiovascular disease: acute myocardial infarction, hypertension and stroke; allergic disease: bronchial asthma, allergy and food allergy; psychiatric disorders: depression, panic disorder, alcohol disorders and drug abuse; cancer, diabetes mellitus, lung disorders, liver disease, epilepsy, tuberculosis, infectious diseases, immune diseases, peptic ulcer disease, renal disease, rheumatoid arthritis, musculoskeletal diseases, eye disorders and ear-nose-throat disorders | Age, BMI, sex, level of education,  smoking status, alcohol consumption, physical activity and co-morbidity |
| Wang et al. (2017) a | 18-59 (42.6) | 17320 | 52.0 | Sleep duration: short (<7h), normal (7-9h), long (>9h) | Anemia, diabetes, hyperlipidemia, cataract/glaucoma, hypertension, ischemic heart disease, cerebrovascular disease, nasopharyngitis, chronic obstructive pulmonary disease, chronic gastroenteritis/peptic ulcer, liver diseases, chronic cholecystitis/gallstones, arthritis, chronic low back pain, chronic nephritis, and urolithiasis | Sociodemographics, lifestyle factors, mental health |
| Wang et al. (2017) b | 60-79 | 4115 | 49.0 | Sleep duration: short (<7h), normal (7-8h), long (>8h) | Anemia, diabetes, hyperlipidemia, cataract/glaucoma, hypertension, ischemic heart disease, cerebrovascular disease, nasopharyngitis, chronic obstructive pulmonary disease, chronic gastroenteritis/peptic ulcer, liver diseases, chronic cholecystitis/gallstones, arthritis, chronic low back pain, chronic nephritis, and urolithiasis | Age, gender, marital status, residence place (rural/urban), education, income, profession, smoking, drinking, fruit intake, physical exercise, BMI, mental health status |
| Zhang et al. (2019) | 35-74 (52.5) | 12765 | 38.0 | Sleep duration: short (≤6h), reference (8h), long (≥9h)  Snoring: never, sometimes, usually  Difficulty falling asleep: yes, no  Early morning arousal: yes, no  Using hypnotics regularly: yes, no | Diabetes, acute myocardial infarction, angina, stroke, including cerebral infarction and cerebral hemorrhage, hypertension, lung, rheumatic heart disease, pulmonary tuberculosis, emphysema, chronic bronchitis, chronic obstructive pulmonary disease, asthma, chronic hepatitis/cirrhosis of the liver, gastrointestinal ulcers, gallstones, cholecystitis, chronic kidney disease, osteoporosis, fractures, rheumatoid arthritis, neurasthenia | BMI, age, education, marital status, annual household income, drinking, smoking, passive smoking and physical activity |

**Abbreviations:** BMI, Body Mass Index; OSA, Obstructive Sleep Apnea; RLS, Restless Legs Syndrome; NA, not available.

**Table S7** The quality assessment for cross-sectional studies.

| Study | Item | | | | | | | | | | | Total score |
| --- | --- | --- | --- | --- | --- | --- | --- | --- | --- | --- | --- | --- |
|  | 1) | 2) | 3) | 4) | 5) | 6) | 7) | 8) | 9) | 10) | 11) |  |
| Appleton et al. (2018) | 1 | 0 | 1 | 1 | 1 | 1 | 0 | 1 | 1 | 0 | 0 | 7 |
| Helbig et al. (2017) | 1 | 0 | 1 | 1 | 1 | 1 | 0 | 1 | 0 | 1 | 1 | 8 |
| Lacedonia et al. (2018) | 1 | 1 | 1 | 1 | 0 | 1 | 0 | 0 | 0 | 0 | 0 | 5 |
| Lima et al. (2012) | 1 | 1 | 1 | 1 | 0 | 0 | 0 | 1 | 1 | 1 | 0 | 7 |
| Liu et al. (2020) | 1 | 1 | 0 | 1 | 0 | 1 | 1 | 0 | 1 | 0 | 0 | 6 |
| Nicholson et al. (2020) | 1 | 1 | 0 | 1 | 0 | 0 | 1 | 1 | 1 | 1 | 0 | 7 |
| Reis et al. (2018) | 1 | 1 | 1 | 1 | 0 | 1 | 0 | 1 | 0 | 0 | 0 | 6 |
| Robichaud-Hallé et al. (2018) | 1 | 1 | 1 | 1 | 0 | 0 | 1 | 1 | 1 | 1 | 1 | 9 |
| Ruel et al. (2018) | 1 | 1 | 1 | 1 | 0 | 1 | 1 | 1 | 1 | 0 | 0 | 8 |
| Ruiz-Castell et al. (2019) | 1 | 1 | 1 | 1 | 0 | 1 | 1 | 1 | 1 | 0 | 0 | 8 |
| Stewart et al. (2006) | 1 | 0 | 1 | 1 | 0 | 0 | 0 | 1 | 0 | 1 | 0 | 5 |
| Torzsa et al. (2011) | 1 | 0 | 1 | 1 | 1 | 1 | 0 | 1 | 0 | 0 | 0 | 6 |
| Wang et al. (2017) a | 1 | 1 | 1 | 1 | 0 | 1 | 0 | 1 | 0 | 1 | 0 | 7 |
| Wang et al. (2017) b | 1 | 0 | 1 | 1 | 0 | 1 | 0 | 1 | 0 | 1 | 0 | 6 |
| Zhang et al. (2019) | 1 | 0 | 0 | 1 | 0 | 1 | 0 | 1 | 0 | 0 | 0 | 4 |

**Table S8** The quality assessment for cohort studies.

| Study | Quality score | | | Total score |
| --- | --- | --- | --- | --- |
|  | Selection | Comparability | Outcome |  |
| He et al. (2021) | 3 | 2 | 1 | 6 |
| Szentkirályi et al. (2014) | 2 | 2 | 1 | 5 |

**Table S9** Associations between abnormal sleep duration and some commonly included chronic conditions in the definition of multimorbidity.

| Chronic conditions | No. of studies | OR (95% CI) | *I^2^*, % | *P* Value for Q test |
| --- | --- | --- | --- | --- |
| Short sleep duration |  |  |  |  |
| Hypertension | 4 | 1.12 (0.98, 1.27) | 47 | 0.13 |
| Diabetes | 4 | 1.21 (1.00, 1.45) | 53 | 0.10 |
| Heart disease | 4 | 1.34 (1.21, 1.49) | 0 | 0.83 |
| Long sleep duration |  |  |  |  |
| Hypertension | 4 | 1.12 (0.96, 1.30) | 34 | 0.21 |
| Diabetes | 4 | 1.15 (0.94, 1.40) | 27 | 0.25 |
| Heart disease | 4 | 1.47 (1.09, 1.98) | 71 | 0.01 |

**Notes:** Odds Ratios (ORs) with 95% confidence intervals (CIs) for multimorbidity in individuals with abnormal sleep duration versus individuals with normal sleep duration are from Lima et al. (2021), Reis et al. (2018), Wang et al. (2017) a and Wang et al. (2017) b. *I^2^* and P value for Q test are used to evaluate the between-study heterogeneity.

Sleep duration categories (short, normal, long): Lima et al. (2021) – ≤6h, 7-8h, ≥9h; Reis et al. (2018) – ≤5h, 6-8h, >9h; Wang et al. (2017)a – <7h, 7-9h, >9h; Wang et al. (2017)b – <7h, 7-8h, >8h

**(A)**
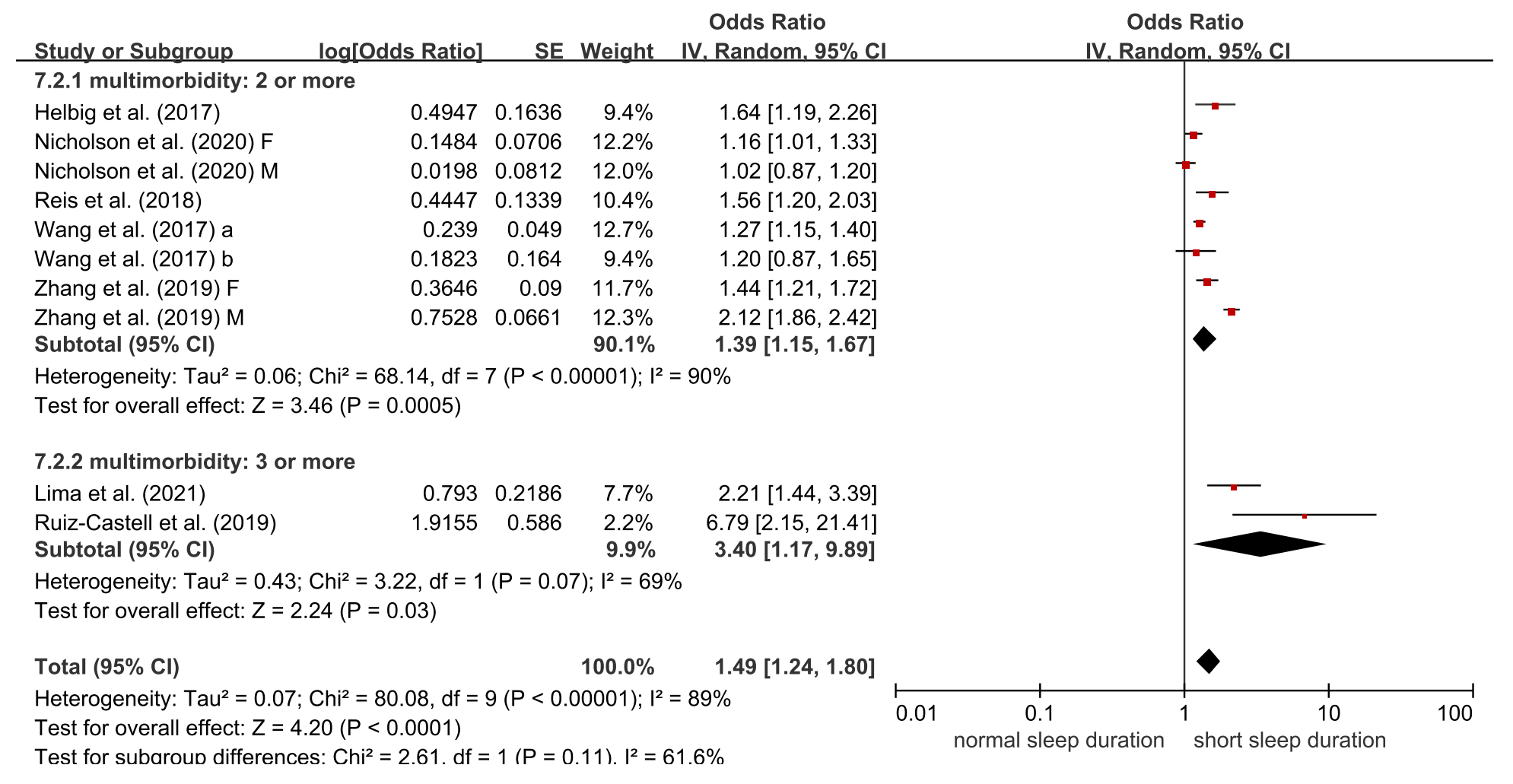


**(B)**
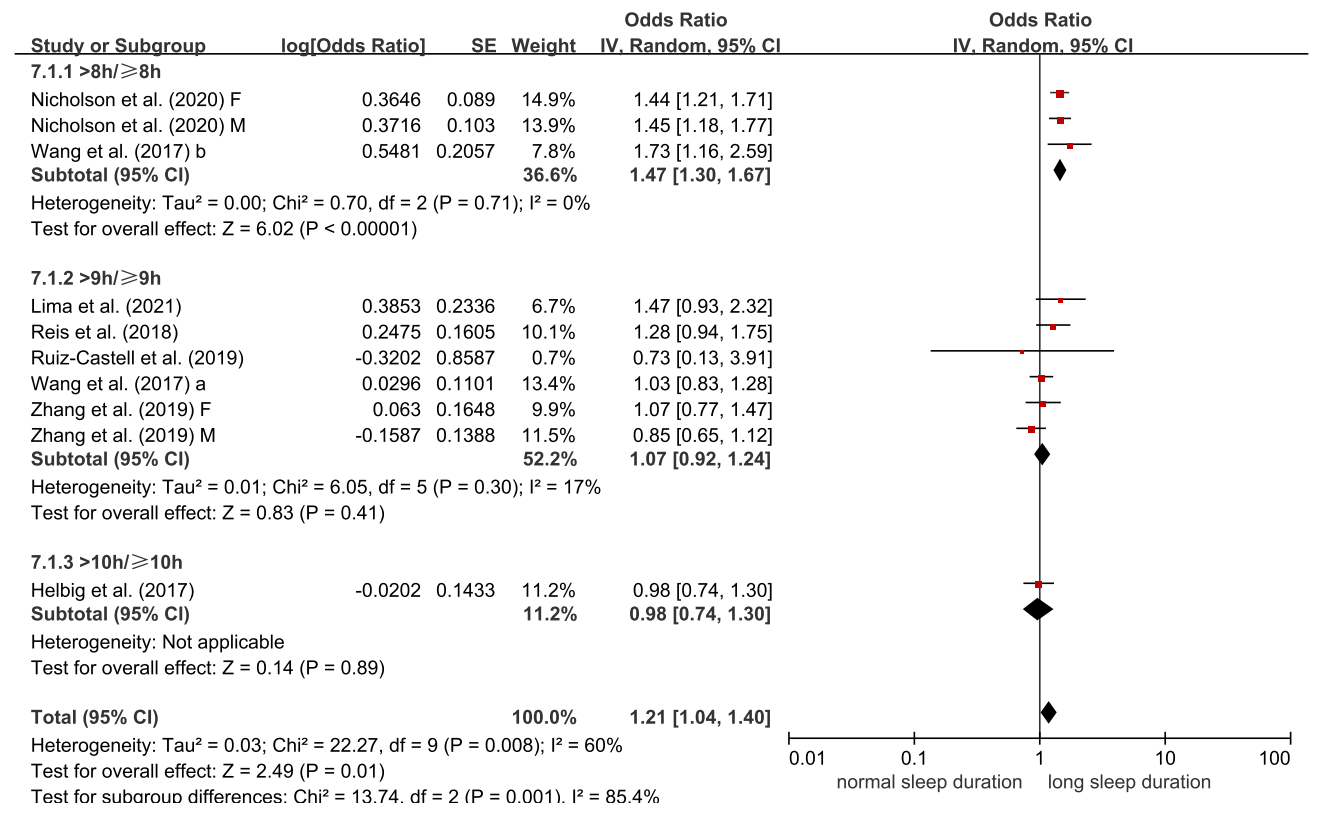


**Figure S1** Meta-analysis of the association between sleep duration and multimorbidity from cross-sectional studies. (A) Short sleep duration and multimorbidity, stratified by the definition of multimorbidity; (B) long sleep duration and multimorbidity, stratified by the definition of long sleep duration.

Normal sleep duration: Helbig et al. (2017) –7-8h; Nicholson et al. (2020) –6-8h; Reis et al. (2018) –6-8h; Zhang et al. (2019) – 8h; Wang et al. (2017) a –7-9h; Wang et al. (2017) b –7-8h; Lima et al. (2021) – 7-8h; Ruiz-Castell et al. (2019) – 6-9h.


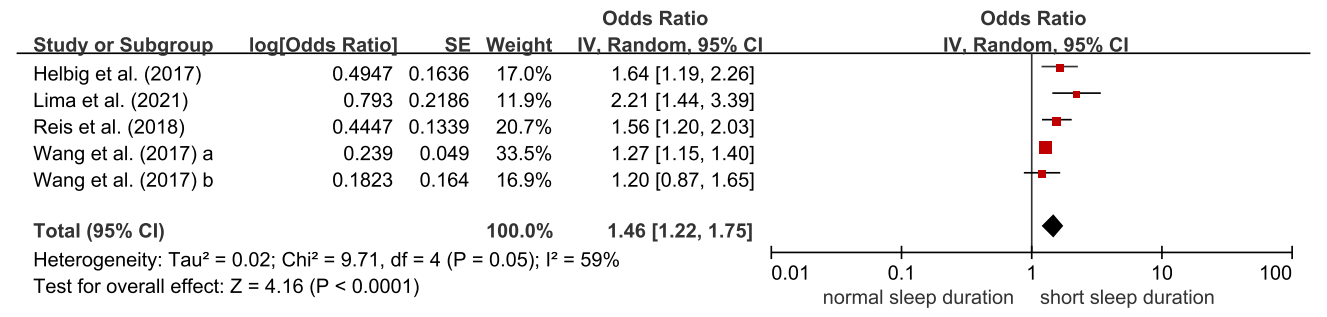


**Figure S2** Meta-analysis of the association between short sleep duration and multimorbidity after removing three outliers.

**(A)**


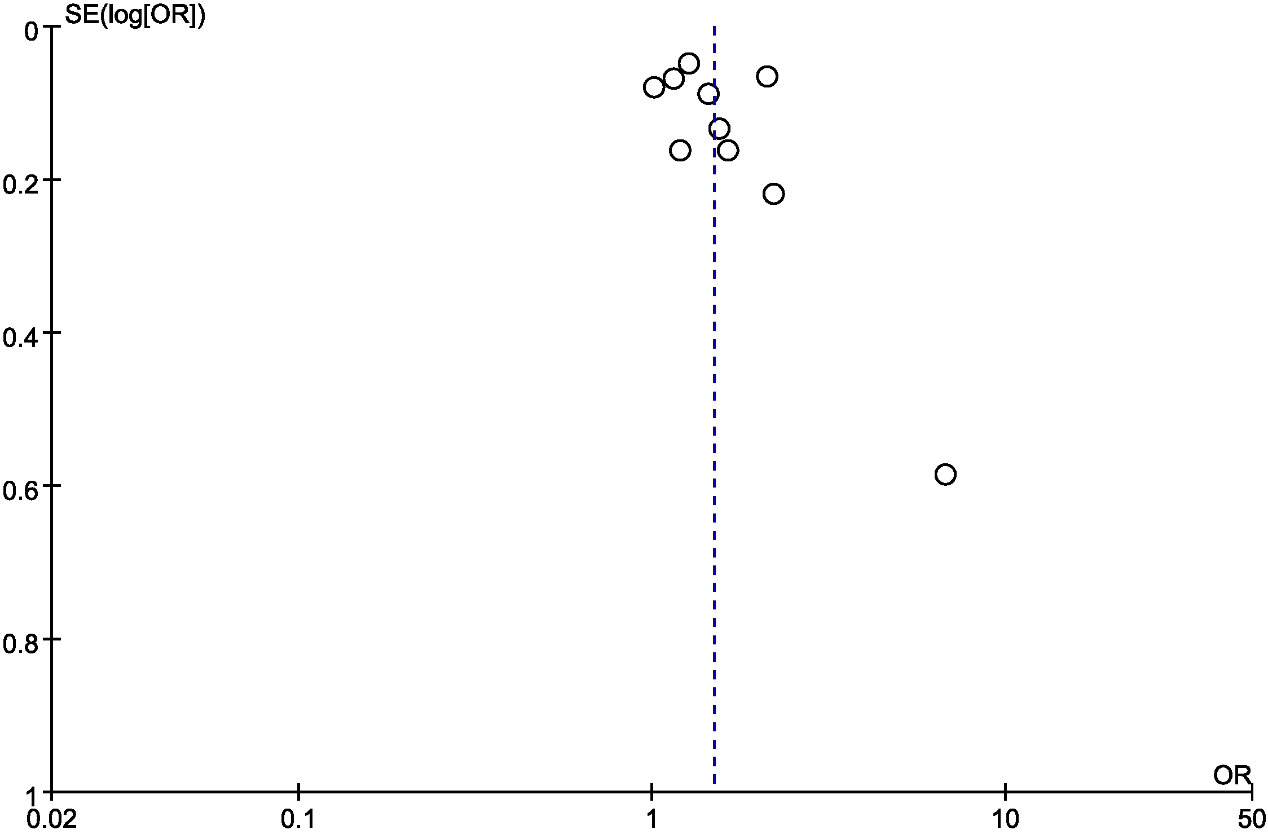


**(B)**
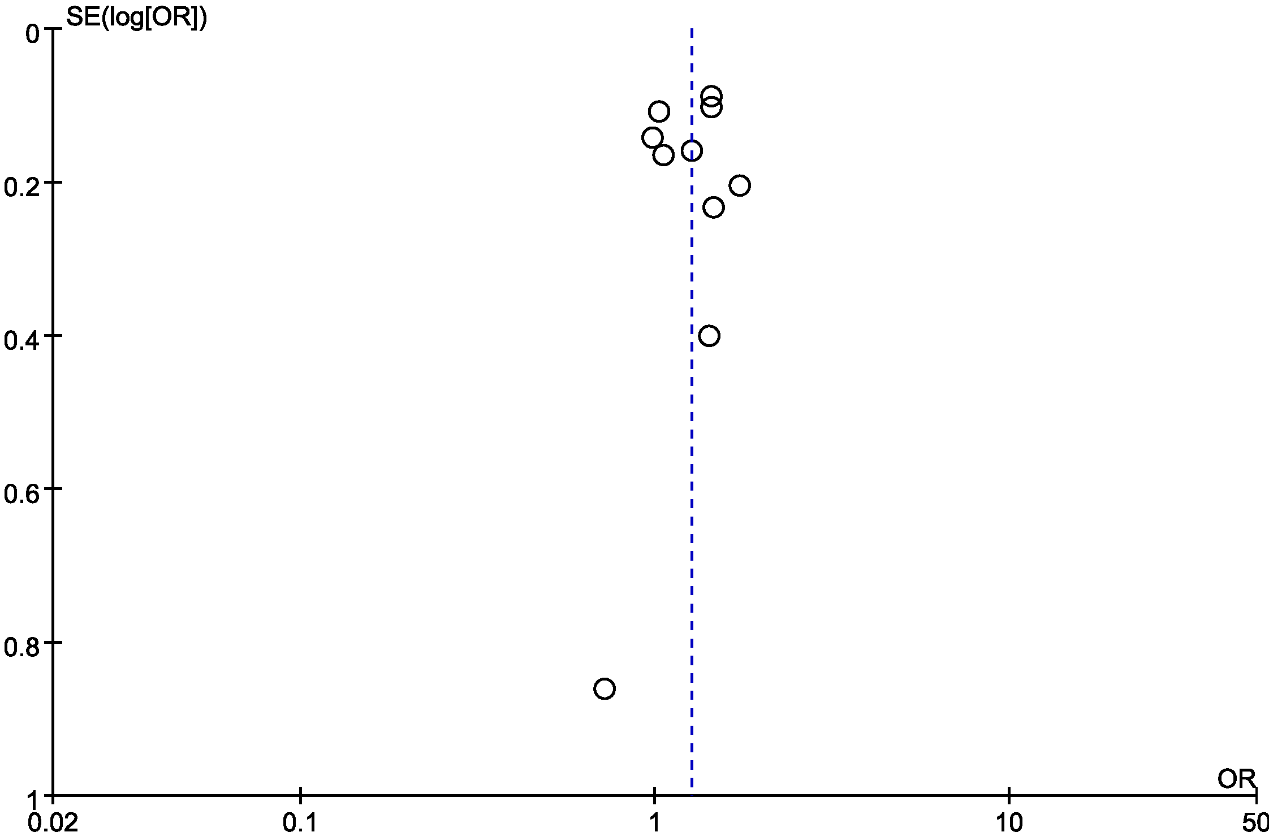


**Figure S3** Funnel plot of studies on abnormal sleep duration and multimorbidity, with the odds ratio on the horizontal axis, and the standard error on the vertical axis. (A) short sleep duration; (B) long sleep duration.


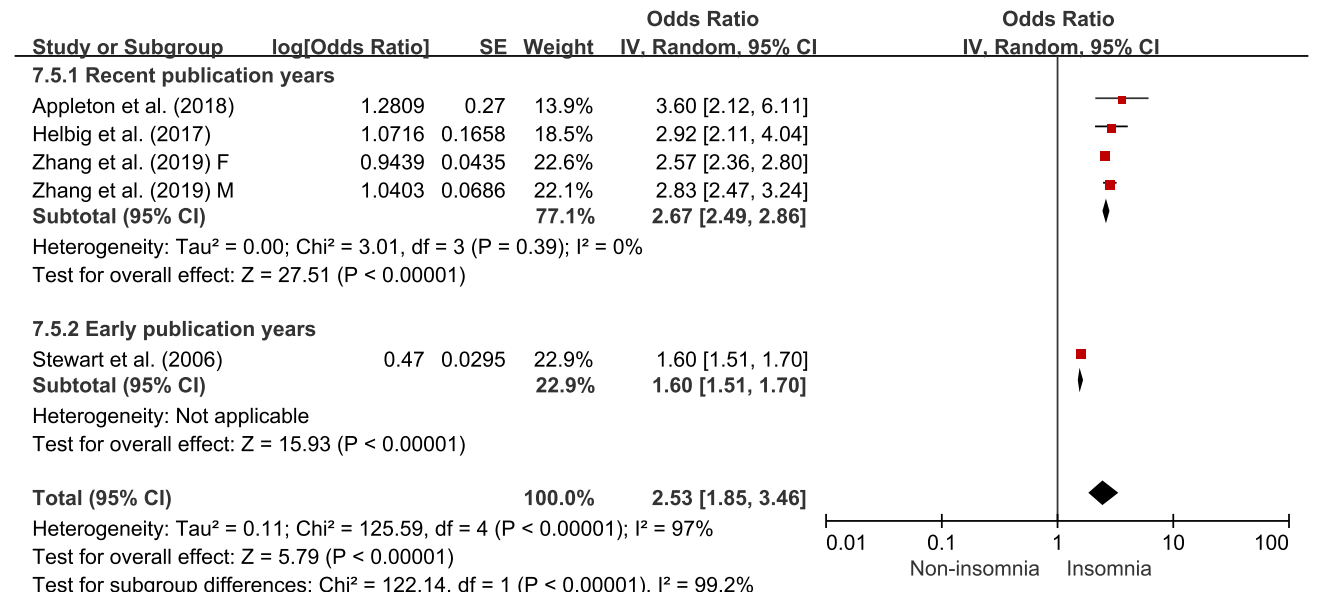


**Figure S4** Meta-analysis of the association between insomnia and multimorbidity from cross-sectional studies, stratified by publication years.


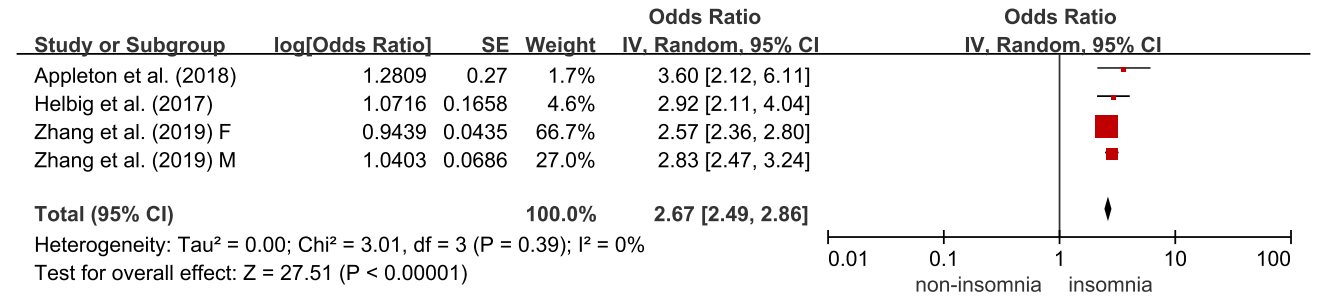


**Figure S5** Meta-analysis of the association between insomnia and multimorbidity after removing one outlier.


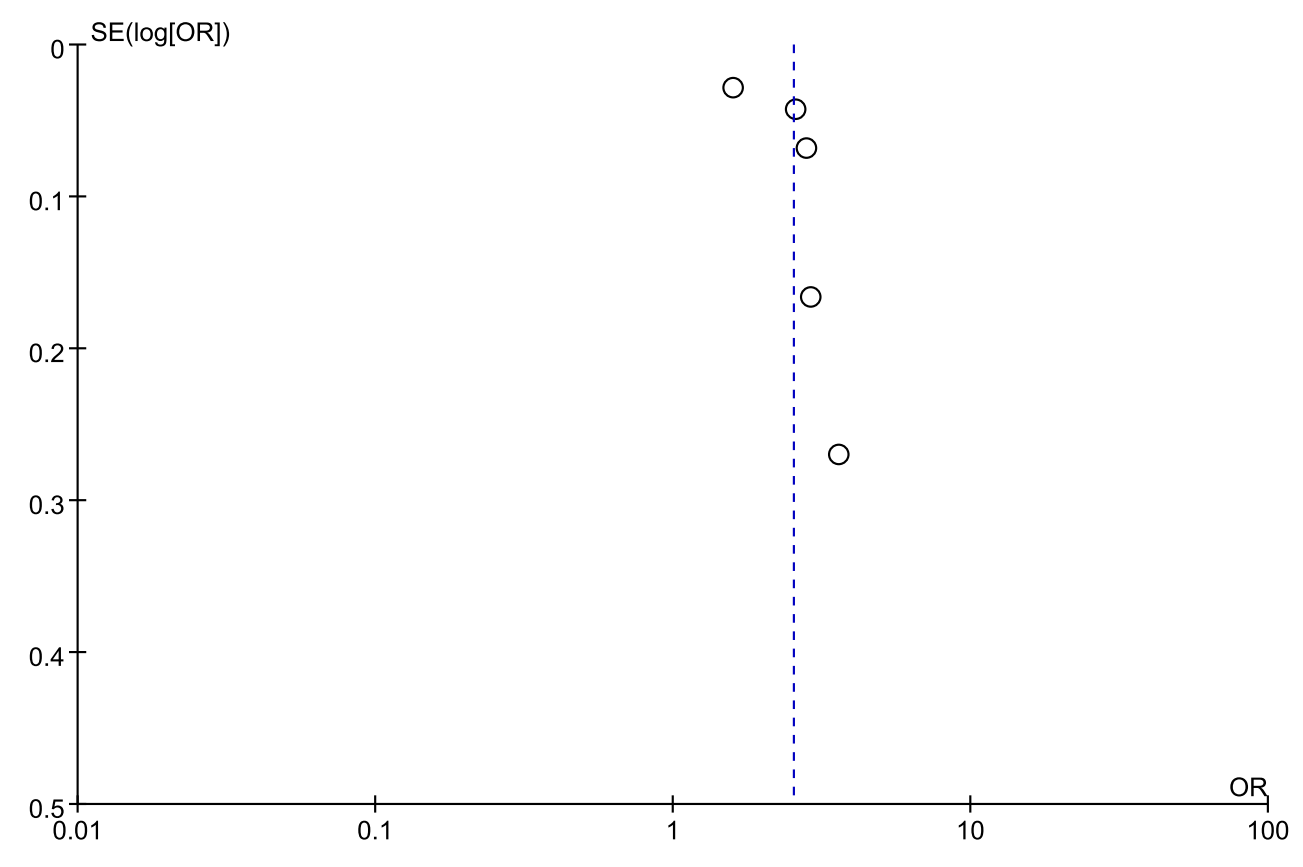
**Figure S6** Funnel plot of studies on insomnia and multimorbidity, with the odds ratio on the horizontal axis, and the standard error on the vertical axis.
